# Asymmetric Structural Engineering of Hot‐Exciton Emitters Achieving a Breakthrough in Non‐Doped BT.2020 Blue OLEDs with a Record 9.5% External Quantum Efficiency

**DOI:** 10.1002/advs.202407254

**Published:** 2024-08-20

**Authors:** Bingzhu Ma, Baijun Zhang, Han Zhang, Yu Huang, Lu Liu, Baoling Wang, Dezhi Yang, Dongge Ma, Ben Zhong Tang, Zhiming Wang

**Affiliations:** ^1^ State Key Laboratory of Luminescent Materials and Devices Guangdong Provincial Key Laboratory of Luminescence from Molecular Aggregates South China University of Technology (SCUT) Guangzhou 510640 China; ^2^ Department of Chemistry, Department of Chemical and Biological Engineering and the Hong Kong Branch of Chinese National Engineering Research Center for Tissue Restoration and Reconstruction The Hong Kong University of Science and Technology Hong Kong 999077 China; ^3^ Intellectual Property Publishing House Co., Ltd No. 50, Meteorological Road, Haidian Beijing 100081 China; ^4^ Center for Aggregation‐Induced Emission, AIE Institute South China University of Technology Guangzhou 510640 China; ^5^ School of Science and Engineering, Shenzhen Institute of Aggregate Science and Technology The Chinese University of Hong Kong Shenzhen (CUHK‐Shenzhen) Guangdong 518172 China

**Keywords:** BT.2020, deep‐blue, hot‐exciton, non‐doped, organic light‐emitting diodes

## Abstract

High‐efficiency non‐doped deep‐blue organic light‐emitting diodes (OLEDs) meeting the standard of BT.2020 color gamut is desired but rarely reported. Herein, an asymmetric structural engineering based on crossed long‐short axis (CLSA) strategy is developed to obtain three new deep‐blue emitters (BICZ, PHDPYCZ, and PHPYCZ) with a hot‐exciton characteristic. Compared to 2BuCz‐CNCz featuring a symmetric single hole‐transport framework, these asymmetric emitters with the introduction of different electron‐transport units show the enhancement of photoluminescence efficiency and improvement of bipolar charge transport capacity. Further combined with high radiative exciton utilization efficiency and light outcoupling efficiency, the non‐doped OLED based on PHPYCZ exhibits the best performance with an excellent current efficiency of 3.49%, a record‐high maximum external quantum efficiency of 9.5%, and a CIE y coordinate of 0.049 approaching the BT.2020 blue point. The breakthrough obtained in this work can inspire the molecular design of deep‐blue emitters for high‐performance non‐doped BT.2020 blue OLEDs.

## Graphical abstract

Deep‐blue hot‐exciton emitters designed by asymmetric long‐short axis strategy enable a good balance of radiative exciton utilization efficiency, photoluminescence efficiency, carrier transport capacity, light outcoupling efficiency, and emission color. The best device exhibits a record‐high maximum external quantum efficiency of 9.5% and CIE y coordinate of 0.049, achieving a breakthrough for non‐doped BT.2020 blue OLEDs.

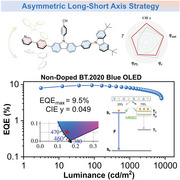

## 1 Introduction

Organic light‐emitting diodes (OLEDs) are considered as a promising class of display technology owing to their self‐illuminating and flexible characteristics.^[^ ^1^ ^]^ As one of the three primary colors, the efficient deep‐blue emitters can reduce the power consumption and broaden the display color gamut of OLEDs, and also be used as host material in emitting layer (EML).^[^ ^2^ ^]^ However, the inherent broadband gap of deep‐blue emitters is not conducive to the charge injection and the carrier combination during the electroluminescence (EL) process.^[^ ^3^ ^]^ As a result, the device performances of deep‐blue emitters lag behind that of red and green emitters, especially the emissions that are close to the Broadcast Service Television 2020 (BT.2020) blue point with CIE_x, y_ = (0.131, 0.046).^[^ ^4^ ^]^ Besides, compared with doped devices that usually face the problem of phase separation and insufficient energy transfer, the non‐doped OLEDs have the advantages of simple process and high stability.^[^ ^5^ ^]^ However, efficient and non‐doped deep‐blue OLEDs meeting the BT.2020 standard are rarely reported so far.

Although phosphorescent emitters and thermally activated delayed fluorescence (TADF) emitters could achieve nearly 100% radiative exciton utilization efficiency (*ƞ*_r_), there are strong intermolecular interaction in metal complexes and the dipole‐dipole interaction in donor–acceptor (D–A) structures, which could result in a significant spectral redshift and severe concentration quenching in aggregates.^[^ ^6^ ^]^ Therefore, these two types of deep‐blue emitters usually require complex doping methods to weaken or eliminate the negative effects of aggregation. Thus, achieving high‐efficiency non‐doped BT.2020 blue OLEDs faces enormous challenges and requires new conceptual developments in molecular design.

Recently, some pure organic deep‐blue emitters with a hot‐exciton characteristic, which possess a good balance between short‐wavelength emission and high exciton utilization, have attracted immense research interest.^[^ ^7^ ^]^ In addition, these hot‐exciton emitters could avoid the occurrence of T_1_ excitons accumulation and annihilation through the rapid high‐lying reverse intersystem crossing (RISC) process (T_n_→S_1_, n ≥ 2), which is well suited for use in non‐doped OLEDs and thus simplify the device preparation.^[^ ^8^ ^]^ For example, Xue et al. designed two phenanthroimidazole‐based deep‐blue emitters with hot‐exciton characteristic, and the EL performance of PIPDMePBO exhibited the best result for non‐doped deep‐blue OLED at that time with a maximum external quantum efficiency (*ƞ*_ext_) of 8.0% and small CIE y coordinate value (0.048) approaching BT.2020 blue point.^[^ ^9^ ^]^ Wang and Liu et al. reported four V‐shaped hot‐exciton emitters based on 2*H*‐imidazole core, and the non‐doped deep‐blue OLED based on FIP‐CZ furnished a high maximum *ƞ*_ext_ of 9.5%.^[^ ^10^ ^]^ Wang and Ma et al. reported two anthracene‐based deep‐blue emitters, and the non‐doped deep‐blue OLED based on 2M‐ph‐*p*CzAnBzt gave a record‐high maximum *ƞ*_ext_ of 10.44%.^[^ ^11^ ^]^ However, their CIE y coordinate values (0.059 and 0.057, respectively) are somewhat deviated from the BT.2020 blue point. For years our group proposed a crossed long‐short axis (CLSA) molecular design strategy for hot‐exciton deep‐blue emitters with a hybridized locally excited (LE) and charge‐transfer (CT) (HLCT) characteristic, where the LE long‐axis skeleton is constructed by connecting the chromophores with small torsion angles, while an orthogonal D–A structure is formed by introducing an electron‐withdrawing group as the short‐axis skeleton to obtain the CT component.^[^ ^12^ ^]^ Based on CLSA strategy, the hot‐exciton emitters 2BuCz‐CNCz^[^ ^13^ ^]^ and 2MCz‐CNMCz^[^ ^14^ ^]^ were designed, and the corresponding non‐doped devices furnished maximum *ƞ*_ext_ values of 5.24% and 7.76% with CIE y coordinates of 0.050 and 0.039, respectively. However, it is worth noting that the hot‐exciton emitters via the CLSA design in our previous work mostly exhibited photoluminescence (PL) efficiency (*ƞ*_PL_) of <80% in toluene. In addition, the chromophores in long‐axis skeleton mainly adopted the carbazole derivatives and showed hole‐dominated carrier transport capacity. Therefore, their EL performances still have much room for improvement.

**Figure 1:**
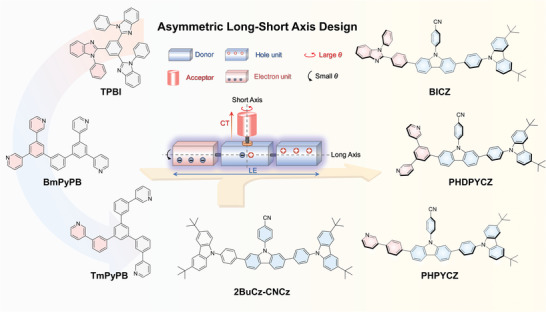
Proposed asymmetric long‐short axis design on hot‐exciton emitters and chemical structures of BICZ, PHDPYCZ, and PHPYCZ.

In view of these, introducing rigid electron transport units through asymmetric modification to enhance structural rigidity and improve electron transport capacity may lead to gain effects. As a validation of hypothesis, three new hot‐exciton deep‐blue emitters, abbreviated BICZ, PHDPYCZ, and PHPYCZ, with asymmetric long‐short axis skeleton were designed and synthesized (**Figure** **1**). And their electron‐transport units were chosen from three classical electron‐transport‐layer (ETL) materials (TPBi, BmPyPB, and TmPyPB), which are different from the reported 2BuCz‐CNCz with a symmetric single hole‐transport framwork. Excitingly, all the three molecules designed by asymmetric structural engineering show high *ƞ*_PL_ up to ≈100% in toluene and improved bipolar charge transport capacity. Furthermore, benefiting from a better balance of radiative exciton utilization efficiency, photoluminescence efficiency, carrier transport capacity and emission color, the non‐doped device based on PHPYCZ achieved a record‐high maximum *ƞ*_ext_ of 9.5% with CIE coordinate of (0.154, 0.049), representing the current state‐of‐the‐art performance for non‐doped deep‐blue OLED approaching the BT.2020 blue point. Meanwhile, thanks to the hot‐exciton process, the device showed small roll‐off in efficiency and kept an *ƞ*_ext_ of 8.05% at brightness of 1000 cd m^−2^.

## 2 Results and Discussion

The synthetic route of BICZ, PHDPYCZ, and PHPYCZ are shown in Scheme S1 (Supporting Information). The intermediate Br‐CNCz‐BuCz was prepared according to the previously reported method,^[^ ^13^ ^]^ and the target molecules were synthesized via Suzuki coupling reaction in a high yield. The chemical structures were confirmed by ^1^H NMR and high‐resolution mass spectrometry (HRMS). The thermal properties of three molecules were evaluated by thermogravimetric analysis (TGA) and differential scanning calorimetry (DSC). The decomposition temperatures (*T*_d_, 5% weight loss) of three molecules are in the range of 489–533 °C. The glass transition temperatures (*T*_g_) of BICZ and PHDPYCZ are above 200 °C, while *T*_g_ is not observed for PHPYCZ, which reflects their good thermal stability (Figure S1, Supporting Information). The electrochemical property of three molecules was investigated via cyclic voltammetry (CV) measurements (Figure S2, Supporting Information). The ionization potentials (IP_CV_) of BICZ, PHDPYCZ, and PHPYCZ were calculated as 5.38, 5.37, and 5.40 eV, which can be approximated as (minus) the energy of the highest occupied molecular orbital (HOMO) level. Meanwhile, the electron affinities (EA_CV_) energy level of BICZ, PHDPYCZ, and PHPYCZ are calculated as 2.38, 2.35, and 2.37 eV, which can be approximated as (minus) the energy of the lowest unoccupied molecular orbital (LUMO) level.

The single crystal of PHPYCZ was obtained by vapor deposition, and its structure and stacking pattern were analyzed (Figure S3, Supporting Information). The crystal structure reveals that the electronic and hole units on the long‐axis skeleton are connected with the central carbazole at smaller torsion angles (*θ*_1_, *θ*_2_) of 28.82° and 40.68°, respectively. While a larger torsion angle (*θ*_3_) of 52.07° between the central carbazole and benzonitrile group (short‐axis skeleton) is observed which is consistent with the long‐short axis skeleton model. Besides, PHPYCZs are stacked in a slipped π‐stacked fashion with a π‐π distance of ≈3.872 Å (Figure S3, Supporting Information). The weak π‐π interaction could help inhabit the concentration quenching and exciton annihilation and thus achieve a high photo‐ and electro‐luminescence efficiency in solid‐states.^[^ ^15^ ^]^

**Figure 2:**
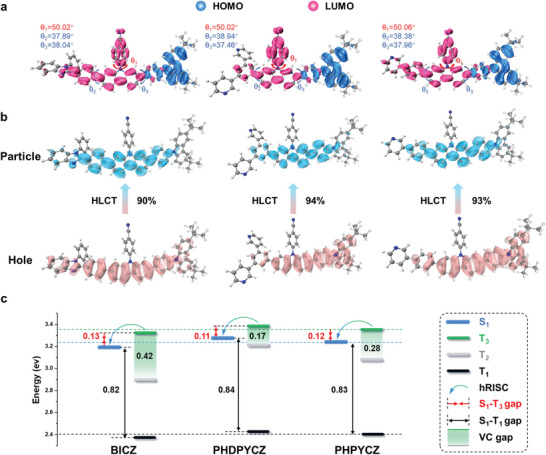
a) Optimized S_0_ geometry structure and HOMO/LUMO distributions. b) NTOs of S_0_→S_1_. c) Proposed hot‐exciton process of BICZ, PHDPYCZ, and PHPYCZ, respectively.

**Figure** **2**a shows optimized ground state (S_0_) structures and the distributions of the frontier molecular orbitals (FMOs) of BICZ, PHDPYCZ, and PHPYCZ. On the long‐axis skeleton of the molecules, both the twisting angles (*θ*_1_, *θ*_2_) between the electron and hole units and the central carbazole are relatively small with values <40°, whereas the benzonitrile group is connected to the central carbazole with a large twist angle (*θ*_3_) of ≈50°. The HOMOs are distributed on the electron‐rich *di*‐*tert*‐butylcarbazole as the hole‐injection groups, while the LUMOs are distributed on central carbazole and the electron units, which are conductive to the electron‐injection. Compared to reported 2BuCz‐CNCz with symmetric structure, the spread LUMO distribution of these asymmetric molecules reflects the improvement in electron transport capacity. Root‐mean‐square displacement (RMSD) was carried out upon S_0_ and the S_1_ geometry of BICZ, PHDPYCZ, and PHPYCZ as well as 2BuCz‐CNCz. Compared to 2BuCz‐CNCz (RMSD: 1.20), BICZ, PHDPYCZ, and PHPYCZ show greater rigidity with small RMSD values of 0.441–0.470 Å (Figure S4, Supporting Information), which may bring an enhancement of *ƞ*_PL_ in solutions.

The natural transition orbitals (NTO) simulation shows that the “hole” and “particle” of BICZ, PHDPYCZ, and PHPYCZ at S_1_ state are dispersed on the long‐axis skeleton of the molecules with large proportion overlap and partial spatial separation (Figure 2b), which corresponds to a LE‐dominant HLCT characteristic. On the other hand, their T_1_ states all show a pure LE‐featured transition with the hole and particle completely overlapped, whereas the high‐lying T_2_ and T_3_ states exhibit the HLCT characteristics similar to the S_1_ state (Figures S6–S8, Supporting Information). In this case, the high‐lying CT components could facilitate the triplet‐to‐singlet conversion from T_2_/T_3_ to S_1_. As a further proof, the energy level diagrams of BICZ, PHDPYCZ, and PHPYCZ were calculated (Figure 2c). It can be seen that BICZ, PHDPYCZ, and PHPYCZ show very similar energy distribution of S_1_, T_1_, and T_3_ states. Their energy gap between S_1_ and T_1_ states (∆*E*_S1T1_) are as large as 0.82–0.84 eV, which make it difficult to induce a sufficient RISC from T_1_ to S_1_. Furthermore, the S_1_/T_3_ energy‐level gaps (∆*E*_S1T3_) are only 0.11–0.13 eV. These small gaps imply the existence of hot‐exciton channels between T_3_ and S_1_, which can harness triplet‐state excitons through the hRISC process. It is worth noting that the internal conversion (IC) process from T_3_ to T_2_ competes with the hRISC process from T_3_ to S_1_, resulting in waste of triplet excitons. In this case, the vibrational coupling (VC) between T_3_ and T_2_ and the hRISC process from T_2_ to S_1_ can alleviate the excitons waste to some extent. It can be seen that the VC gaps between T_2_ and T_3_ are in the order of PHDPYCZ (0.17 eV) < PHPYCZ (0.28 eV) < BICZ (0.42 eV), while the S_1_–T_2_ gap values are also calculated in the order of PHDPYCZ (0.06 eV) < PHPYCZ (0.16 eV) < BICZ (0.29 eV). Besides, the IC process between T_2_ and T_1_ can also lead to the waste of triplet excitons, and the energy level difference between T_2_ and T_1_ were further calculated in the order of PHDPYCZ (0.78 eV) > PHPYCZ (0.68 eV) > BICZ (0.53 eV). These results indicate that the *ƞ*_r_ values of these molecules in EL process should be in the order of PHDPYCZ > PHPYCZ > BICZ.

**Figure 3:**
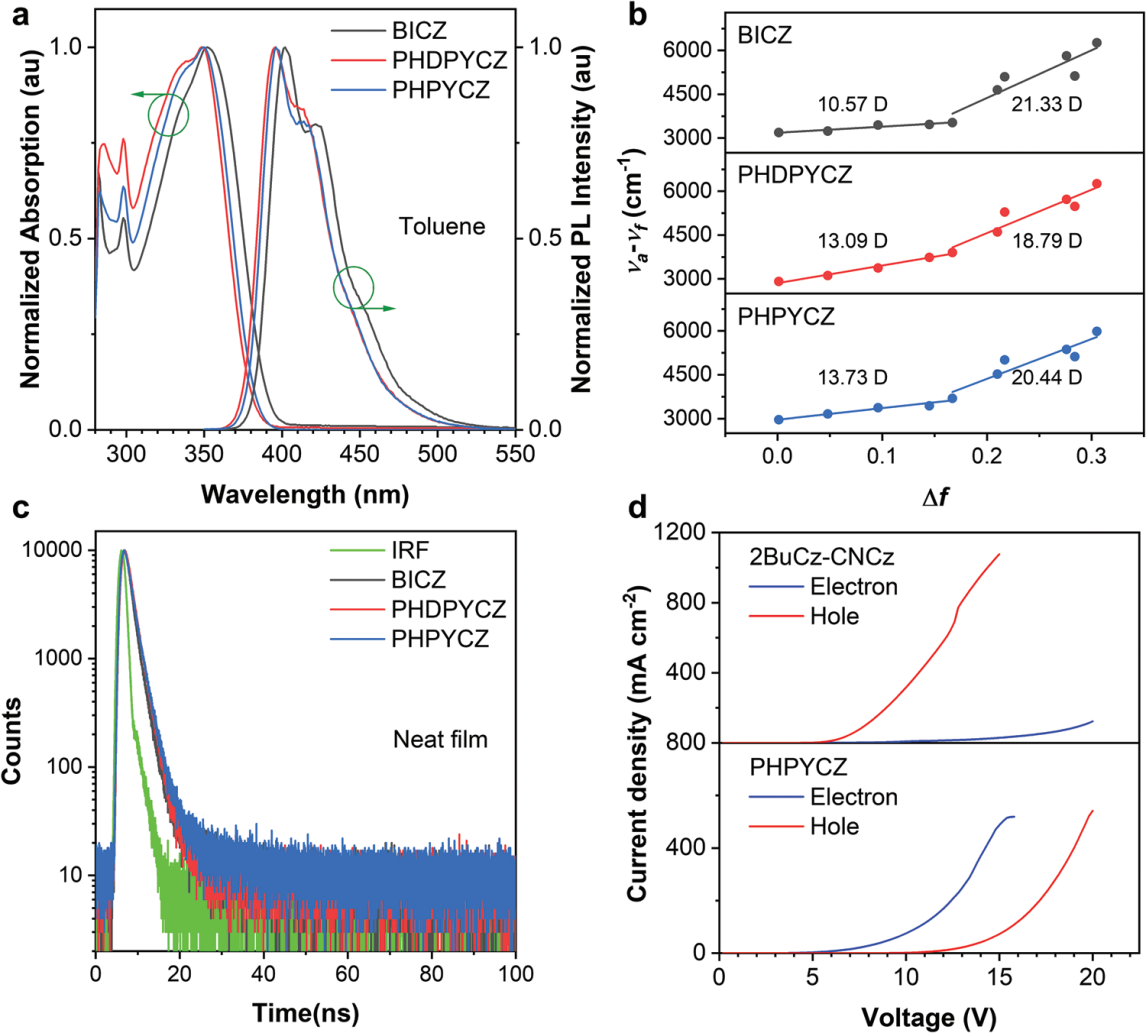
a) UV/Vis absorption and PL spectra in toluene (10^−5^ m). b) Solvatochromic Lippert–Mataga models. c) Transient PL spectra in neat films. d) Plots of current density versus applied voltage of the electron‐only and hole‐only devices based on 2BuCz‐CNCz and PHPYCZ, respectively.

**Figure** **3**a shows the UV‐visible absorption and PL spectra of BICZ, PHDPYCZ, and PHPYCZ in dilute toluene solutions (10^−5^ M), and the detailed data were summarized in **Table** **1**. The absorption band at 298 nm can be ascribed to the π–π* transition of carbazolyl fragment, while the strong and broad absorption band at 350 nm should be caused by the π‐conjugation of the long‐axis skeleton. Under the excitation of a UV lamp, BICZ, PHDPYCZ, and PHPYCZ exhibit deep‐blue emission with emission peaks (*λ*_em_) at 402, 396 and 396 nm, respectively. Compared to the other two emitters, BICZ has a certain degree of conjugation extension that leads to the redshift of the spectrum, which is also clearly observed in the NTO of the S_1_ state. Excitingly, all three molecules exhibit high *ƞ*_PL_ with values up to ≈100% in toluene solution, which is in good agreement with the calculated anticipation, demonstrating the feasibility of the asymmetric long‐short axis design. After fabricated into neat films, the PL spectra of BICZ, PHDPYCZ, and PHPYCZ show red‐shifts to 424, 417, and 417 nm (Figure S11, Supporting Information) with decreased *ƞ*_PL_ values of 77.2%, 71.8%, and 63.6%, respectively, due to the solid‐state polarization and aggregation effect. Nevertheless, their *ƞ*_PL_ values in neat films are still much higher than that of 2BuCz‐CNCz (35.7%), which is beneficial for achieving high‐efficiency non‐doped OLEDs.

**Table 1:** Optical, thermal, and electrochemical properties of BICZ, PHDPYCZ, and PHPYCZ.

Emitters	*λ*_abs_ [nm]	*λ*_PL_ [nm]		*ƞ*_PL_ [%]^a)^		S_1_/T_1_/ ∆*E*_S1T1_ [eV]^d)^	*T*_g_/*T*_d_ [°C]	IP_CV_/EA_CV_ [eV]^e)^
		Soln^a)^	Film^b)^	Soln^a)^	Film^b)^			
BICZ	352	402	424	≈100	77.2	3.31/2.69/0.62	202/527	5.38/2.38
PHDPYCZ	349	396	417	≈100	71.8	3.27/2.61/0.66	200/533	5.37/2.35
PHPYCZ	349	396	417	≈100	63.6	3.32/2.63/0.69	nd/489	5.4/2.37

^a)^ Measured in toluene solution (10^−5^ m);

^b)^ Measured in vacuum‐deposited neat film;

^c)^ Absolute photoluminescence quantum efficiency;

^d)^ Estimated from the onsets of the fluorescence (298 K) and phosphorescence spectra (77 K) in toluene. *ΔE*_S1T1_ = *E*_S1_ − *E*_T1_;

^e)^ IP_CV_ and EA_CV_ were measured from the oxidation potential by cyclic voltammetry with ferrocene as the external standard; nd = not detectable.

To investigate the lowest singlet (S_1_) excited state of BICZ, PHDPYCZ, and PHPYCZ, absorption and PL spectra were measured at different solvents (Figure S9, Supporting Information). As the solvent polarity increases from *n*‐hexane to acetonitrile, the PL spectra of BICZ, PHDPYCZ, and PHPYCZ all are gradually broadened and red‐shifted, accompanied by the disappearance of vibronic structures. Additionally, the Stokes shifts (*v*_a_–*v*_f_) of these emitters show two section linear relations with the solvent orientational polarizability (*f*) (Figure 3b). According to the Lippert–Mataga equation,^[^ ^16^ ^]^ the dipole moment (*u*_e_) values of the S_1_ state are estimated to be 10.57, 13.9, and 13.73 D in low‐polarity solvents (*f* ≤ 0.167) and 21.33, 18.79, and 20.44 D in high‐polarity solvents (*f* ≥ 0.21), manifesting the LE‐featured and CT‐featured radiation transition, respectively.

The S_1_ and T_1_ energy levels of BICZ, PHDPYCZ, and PHPYCZ were estimated by the onsets of room‐temperature fluorescence and low‐temperature phosphorescence spectra in toluene (Table 1; Figure S10, Supporting Information). Their *E*_S1_ and *E*_T1_ distributions are very similar with values of 3.27–3.32 eV and 2.61–2.69 eV, respectively, which is consistent with the calculation results. The large Δ*E*_S1T1_ values of these molecules (0.62–0.69 eV) are hard to induce delayed fluorescence, therefore nanosecond‐scaled lifetimes are observed in their transient PL spectra in both toluene (Figure S11, Supporting Information) and neat film (Figure 3c).

To deeply understand their carriers transport capacity, the electron‐only and hole‐only devices were fabricated with the configuration of ITO/ TmPyPB (10 nm)/EMLs (80 nm)/TmPyPB (10 nm)/LiF (1 nm)/Al (120 nm), and ITO/TAPC (10 nm)/EMLs (80 nm)/TAPC (10 nm)/Al (120 nm), where EMLs are 2BuCz‐CNCz, BICZ, PHDPYCZ, and PHPYCZ, respectively. Figure 3d and Figure S12 (Supporting Information) show their voltage–current density curves. It can be seen that 2BuCz‐CNCz exhibits a single hole‐transport property due to its structure containing only carbazole groups. As expected, BICZ, PHDPYCZ, and PHPYCZ with the introduction of electron transport units indeed improve the electron transport capacity, and their electron‐dominated bipolar charge transport capacity could contribute to the electron–hole charge balance in the EL process and thus improve device performance.

**Figure 4:**
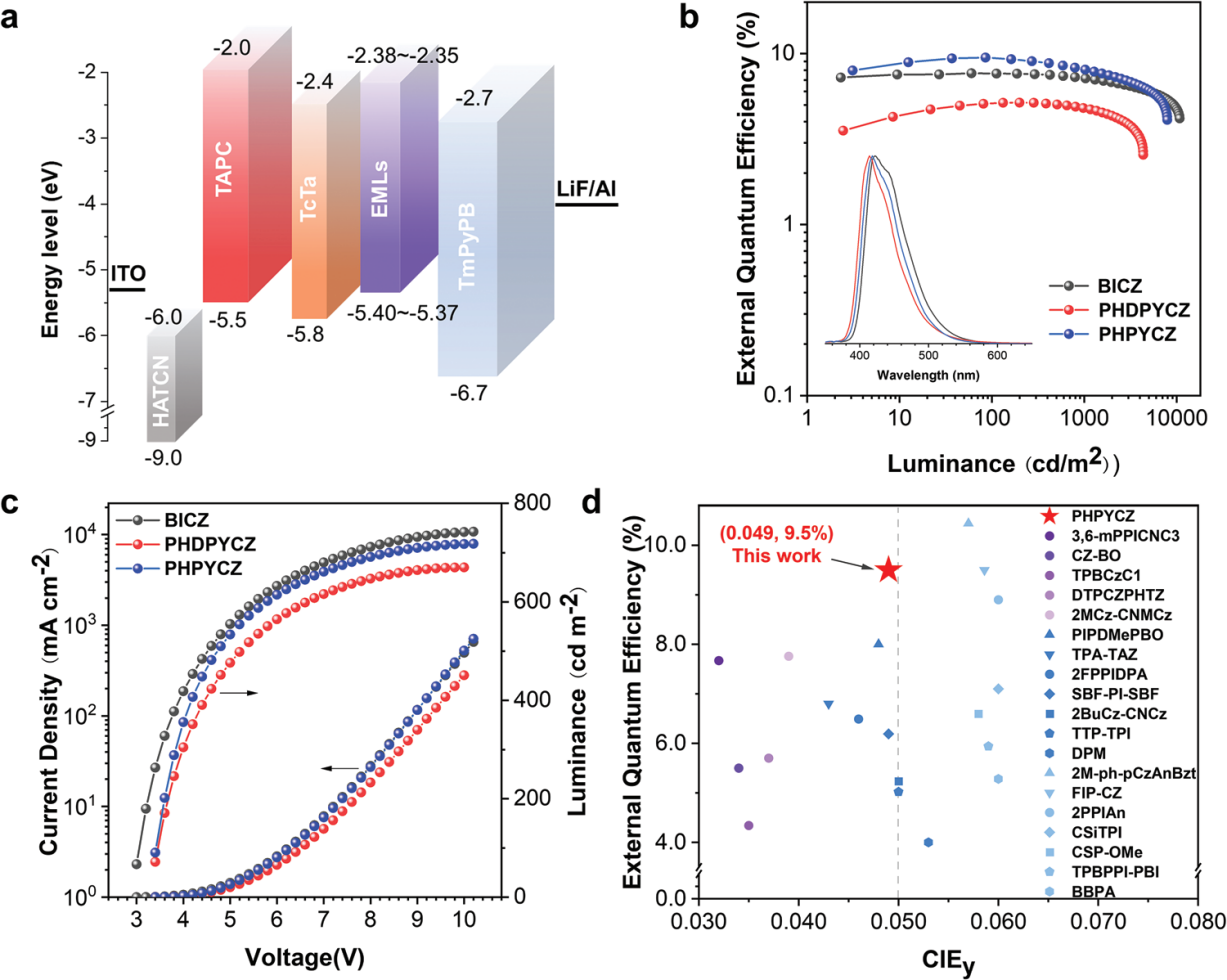
a) Device structure, ionization potentials (IP_CV_) and electron affinities (EA_CV_) for each material. b) External quantum efficiency−luminance curves of the non‐doped OLEDs based on BICZ, PHDPYCZ, and PHPYCZ. Inset shows the EL spectra. c) Current density‐voltage‐luminance (*J–V–L*) characteristics. d) Representative EL performance of reported non‐doped deep‐blue OLEDs with CIE y < 0.06.

To explore the EL properties of BICZ, PHDPYCZ, and PHPYCZ, non‐doped OLEDs with the optimized device architecture of ITO/HATCN (5 nm)/TAPC (25 nm)/TcTa (15 nm)/EMLs (20 nm)/TmPyPB (40 nm)/LiF (1 nm)/Al were fabricated (**Figure** **4**a), where EMLs stands for BICZ, PHDPYCZ, and PHPYCZ. In these devices, ITO (indium tin oxide) and aluminum are adopted as the anode and cathodes, respectively; 1,4,5,8,9,11‐hexaazatriphenylenehexacarbonitrile (HATCN) is a well‐known hole‐injecting material; di‐(4‐(N,N‐ditolyl‐amino)‐phenyl)cyclohexane (TAPC) works as a hole‐transporting layer; tris(4‐carbazoyl‐9‐ylphenyl)amine (TCTA) serves as an exciton‐blocking layer; 3,3′‐[5′‐[3‐(3‐Pyridinyl)phenyl][1,1′:3′,1″‐terphenyl]−3,3″‐diyl]bispyridine (TmPyPB) and lithium fluoride (LiF) function as the ETL and electron‐injection layer. The performances of these non‐doped OLEDs were listed in **Table** **2**. The EL spectra of these devices exhibit deep‐blue emission peaking at 422, 414, and 418 nm, respectively, resembling the PL behaviors of their neat films (Figure 4b). Among them, the non‐doped device based on PHPYCZ exhibits the best EL performance with a maximum luminance (*L*) of 7917 cd m^−2^, a maximum current efficiency (*η*_c_) of 3.49 cd/A, a maximum power efficiency (*η*_p_) of 2.86 lm/W, and a record‐high maximum *ƞ*_ext_ of 9.5% with CIE coordinates of (0.154,0.049). The device also shows a small efficiency roll‐off at brightness of 1000 cd m^−2^ with *ƞ*_ext_ of 8.05%. To our knowledge, the EL performance of PHPYCZ is the best result for non‐doped OLED approaching the BT.2020 blue point (Figure 4d).

**Table 2:** Summary of performance of non‐doped devices.

Non‐dope device	*λ*_EL_ [nm]^a)^	FWHM [nm]^a)^	*V*_on_ [V]^b)^	*L* [cd m^−2^]^c)^	*η*_c_ [cd/A]^c)^	*η*_p_ [lm/W]^c)^	*η*_ext_ [%]^d)^	CIE (x, y)^e)^
BICZ	422	64	3.0	10 780	3.82	3.33	7.67/7.16	(0.153,0.063)
PHDPYCZ	414	58	3.4	4348	1.95	1.47	5.17/4.72	(0.159,0.056)
PHPYCZ	418	58	3.4	7917	3.49	2.86	9.50/8.05	(0.154,0.049)

^a)^ *λ*_EL_ = EL maximum;

^b)^ *V*_on_ = turn‐on voltage at 1 cd m^−2^;

^c)^ Luminescence (*L*), current efficiency (*η*_c_), power efficiency (*η*_p_);

^d)^ External quantum efficiency (*η*_ext_) at maximum and 1000 cd m^−2^;

^e)^ CIE = Commission Internationale de L'Eclairage, recorded at 5 V.

Compared to PHPYCZ, the non‐doped OLEDs based on BICZ and PHDPYCZ have lower EL efficiency with *ƞ*_ext_ of 7.67% and 5.17%, respectively. To investigate the significant *ƞ*_ext_ differences among them, the main parameters contributing to EL efficiency were discussed according to the formula:^[^ ^17^ ^]^

(1) $$\eta_{ext}=\gamma \;\eta_{PL}\eta_{r}\eta_{out}$$

where *γ* is the electron–hole charge balance factor, *ƞ*_out_ is the light outcoupling efficiency, and *ƞ*_PL_ is recorded at vacuum‐deposited films. First, the variable‐angle‐dependent PL spectra of BICZ, PHDPYCZ, and PHPYCZ in neat films were tested (Figure S15, Supporting Information), and they exhibit prefer horizontal dipole orientation with large Θ//s values of 90%, 88%, and 89%, respectively. Performing optical simulation according to the device structure, the *ƞ*_out_ values of BICZ, PHDPYCZ, and PHPYCZ were calculated to be 30%, 28%, and 29% respectively. As mentioned above, the *ƞ*_r_ values of these molecules are in the order of PHDPYCZ > PHPYCZ > BICZ, and *ƞ*_PL_ values of these molecules at vacuum‐deposited neat films are in the order of BICZ (77.2%) > PHDPYCZ (71.8%) > PHPYCZ (63.6%). Thus, considering the similar *ƞ*_out_, highest *ƞ*_r_, and competitive *ƞ*_PL_, PHDPYCZ should have the best device efficiency in theory. However, its experimental result is exactly the opposite, which may be caused by the most unbalanced carrier transport capacity among the three molecules (Figure S13, Supporting Information). On the other hand, despite showing the worst *ƞ*_r_, BICZ has the highest *ƞ*_PL_ as well as the most balanced carrier transport, which leads to its considerable *ƞ*_ext_. Finally, owning a good balance of *ƞ*_r_, *ƞ*_PL_, and carrier transport makes PHPYCZ possess the best EL performance. To our knowledge, this is the first time that the EL effciency of device is comprehensively evaluated from the four parameters in Equation (1).

In order to more accurately analyze the process of effective triplet‐excitons utilization, the transient EL spectra of non‐doped devices based on BICZ, PHDPYCZ, and PHPYCZ were measured (Figure S16, Supporting Information). The delayed component of these devices shows a gradual decrease in the ratio of delayed component as the driving voltage increases from 5 to 9 V, because of the enhanced triplet excitons quenching process at higher driving voltages. Furthermore, the delay curve of the transient EL was fully fitted by the triplet‐triplet annihilation (TTA) model,^[^ ^18^ ^]^ and accordingly, the proportion of delayed fluorescence caused by the TTA process is calculated to be only 3.39% for BICZ, 5.40% for PHDPYCZ, and 8.32% for PHPYCZ. This result could strongly confirm that the rapid RISC process of high triplet energy levels (T_3_ and T_2_) is the main way to utilize triplet excitons in the EL process.

## 3 Conclusion

In summary, three new deep‐blue hot‐exciton emitters, BICZ, PHDPYCZ, and PHPYCZ, were successfully synthesized by asymmetric structural engineering. Experimental and theoretical calculations show that the enhancement of structural rigidity contribute to their high *ƞ*_PL_ of ≈100% in toluene. Meanwhile, BICZ, PHDPYCZ, and PHPYCZ all exhibit the HLCT characteristic, which can facilitate the triplet‐to‐singlet conversion via the hRISC process from T_2_/T_3_ to S_1._ Compared to 2BuCz‐CNCz with single hole‐transport property, BICZ, PHDPYCZ, and PHPYCZ all exhibit electron‐dominated bipolar charge capacity, owing to the asymmetric introduction of electronic transport units. Besides, they exhibit prefer horizontal dipole orientation with large *Θ*//s (90%, 88% and 89%). Among them, PHPYCZ has a good balance of *ƞ*_r_, *ƞ*_PL_, carrier transport capacity as well as emission color, which make its non‐doped device show the best EL performance with a record‐high *ƞ*_ext_ of 9.5% and CIE coordinates of (0.154, 0.049) approaching the BT.2020 blue point. These results demonstrate the feasibility of the asymmetric long‐short axis design and provides effective guidance for the development of high‐performance non‐doped BT.2020 blue OLEDs.

## Conflict of Interest

The authors declare no conflict of interest.

## Supporting information

Supporting Information

## Data Availability

The data that support the findings of this study are available from the corresponding author upon reasonable request.
