# Supplementary material for: Asymmetric Structural Engineering of Hot‐Exciton Emitters Achieving a Breakthrough in Non‐Doped BT.2020 Blue OLEDs with a Record 9.5% External Quantum Efficiency
Source: Adv Sci (Weinh). 2024 Aug 20;11(39):2407254. doi: 10.1002/advs.202407254 (PMC11497023; doi:10.1002/advs.202407254)
Supplement: Supplementary file 1 — Supporting Information [file ADVS-11-2407254-s001.docx]

Supporting Information

Asymmetric Structural Engineering of Hot-Exciton Emitters Achieving a Breakthrough in Non-Doped BT.2020 Blue OLEDs with a Record 9.5% External Quantum Efficiency

Bingzhu Ma, Baijun Zhang, Han Zhang*, Yu Huang, Lu Liu, Baoling Wang, Dezhi Yang, Dongge Ma, Ben Zhong Tang*, and Zhiming Wang*

**Contents**

1. **General Information**
2. **Experimental Section**
3. **Single crystal data**
4. **Computational Methods**
5. **Additional Spectra and Data**
6. **Device fabrication and measurement**
7. **Configuration optimization of non-doped device based on PHPYCZ**
8. **NMR Spectroscopies and Mass spectrums**
9. **General Information**

All the reagents and solvents were purchased from commercial sources and used as received without further purification. The final products were subjected to vacuum sublimation to further improve purity before photoluminescence (PL) and electroluminescence (EL) properties investigations. ^1^H and ^13^C NMR spectra were recorded on a Bruker AV 500 spectrometer in appropriated deuterated dichloromethane at room temperature. High resolution mass spectra (HRMS) were tested on Agilent1290/Bruker maXis impact. MALDI-TOF MS were tested on Bruker autoflex III smartbean. Thermogravimetric analysis (TGA) was performed on a TA TGA Q5000 from 30 ℃ to 800 ℃ under dry nitrogen at a heating rate of 10 °C min^−1^. The differential scanning calorimetry (DSC) analysis was carried out on a DSC Q1000 from 30 ℃ to 300 ℃ under dry nitrogen at a heating rate of 10 °C min^−1^. Cyclic voltammetry (CV) was measured on a CHI 610E A14297 in a solution of tetra-*n*-butylammonium hexafluorophosphate (Bu4NPF6) (0.1 M) in dichloromethane or dimethylformamide at a scan rate of 100 mV s^‒1^, using a platinum wire as the auxiliary electrode, a glass carbon disk as the working electrode and Ag/Ag+ as the reference electrode. Ionization Potential (IP_CV_) = [*E*_ox_ − *E*_1/2_(Fc/Fc^+^) + 4.8] eV, Electron Affinities (EA_CV_) = [*E*_red_ − *E*_1/2_(Fc/Fc^+^) + 4.8] eV, where *E*_ox_ and *E*_red_ represent the onset oxidation potential and the reduction potential relative to Fc/Fc^+^ (4.8 eV), respectively, and *E*_1/2_(Fc/Fc^+^) represents the calibrated value. UV-vis absorption spectra were recorded with a Shimadzu UV-2600 spectrophotometer. Measurements of PL spectra were carried out on Horiba Fluoromax-4 spectrofluorometer. Fluorescence quantum yields in solutions and solid films were measured using a Hamamatsu absolute PL quantum yield spectrometer C11347 Quantaurus QY. Fluorescence lifetimes were determined on an Edinburgh FLS1000 spectrometer.

1. **Experimental Section**
   1. **Synthesis and characterization**


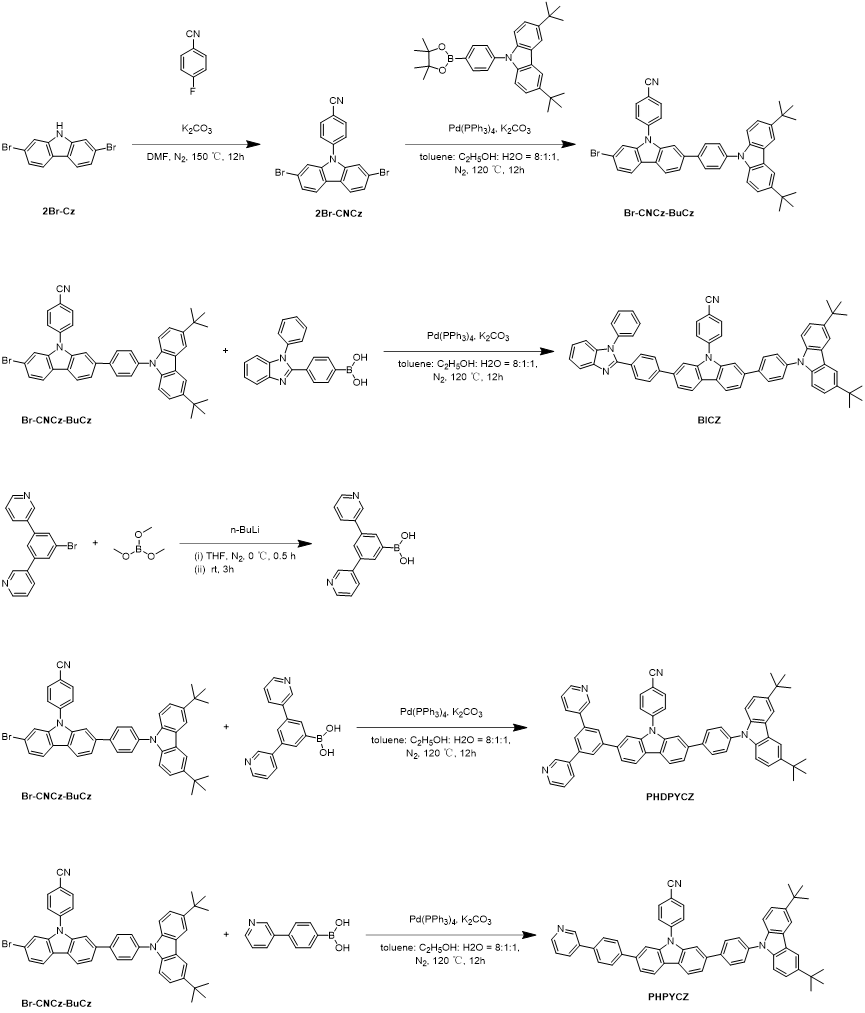


**Scheme S1.** Molecular structures and synthetic routes of the **BICZ**, **PHDPYCZ** and **PHPYCZ**.

**4-(2-bromo-7-(4-(3,6-di-tert-butyl-9H-carbazol-9-yl)phenyl)-9H-carbazol-9-yl)benzonitrile(Br-CNCz-BuCz):** 2Br-CNCz is prepared by referring to the method reported in the literature. A mixture of compound 2Br-CNCz (1.53 g, 3.6 mmol), 3,6-di-tert-butyl-9-(4-(4,4,5,5-tetramethyl-1,3,2-dioxaborolan-2-yl)phenyl)-9H-carbazole (1.44 g, 3 mmol), Pd(PPh3)4 (0.346 g, 0.3 mmol), and potassium carbonate (1.24 g, 9 mmol) was added in 250 mL two-neck bottle under nitrogen. Then, a mixed solvent system of toluene, C_2_H_5_OH and H_2_O (v/v/v = 8:1:1) was injected into the bottle, and the reaction mixture was refluxed for 12 h. After cooling to room temperature, the mixture was poured into water and extracted twice with dichloromethane, and then dried over anhydrous magnesium sulfate. After filtration, the solvent was evaporated under reduced pressure and the residue was purified by silica-gel column chromatography (dichloromethane/petroleum). White solid of Br-CNCz-BuCz was obtained in 60% yield (1.27 g). ^1^H NMR (400 MHz, CDCl_3_), δ (TMS, ppm): δ 8.21 (d, J = 8.0 Hz, 1H), 8.15 (d, J = 1.6 Hz, 2H), 8.01 (dd, J = 16.1, 8.4 Hz, 3H), 7.80 (t, J = 8.8 Hz, 4H), 7.70 – 7.62 (m, 4H), 7.58 (d, J = 1.4 Hz, 1H), 7.47 (dt, J = 8.7, 1.9 Hz, 3H), 7.40 (d, J = 8.6 Hz, 2H), 1.47 (s, 18H). ^13^C NMR (101 MHz, CDCl_3_) δ 143.02, 141.36, 141.30, 140.86, 139.82, 139.59, 139.12, 137.64, 134.31, 128.73, 127.46, 126.99, 124.43, 123.64, 123.47, 122.71, 122.66, 121.81, 121.04, 120.12, 118.11, 116.32, 112.72, 111.46, 109.19, 108.12, 34.75, 32.02.

**4-(2-(4-(3,6-di-tert-butyl-9H-carbazol-9-yl)phenyl)-7-(4-(1-phenyl-1H-benzo[d]imidazol-2-yl)phenyl)-9H-carbazol-9-yl)benzonitrile(BICZ):** A mixture of compound Br-CNCz-BuCz (2.1 g, 3 mmol), (4-(1-phenyl-1H-benzo[d]imidazol-2-yl)phenyl)boronic acid (1.13 g, 3.6 mmol), Pd(PPh3)4 (0.346 g, 0.3 mmol), and potassium carbonate (1.24 g, 9 mmol) was added in 250 mL two-neck bottle under nitrogen. Then, a mixed solvent system of toluene, C_2_H_5_OH and H_2_O (v/v/v = 8:1:1) was injected into the bottle, and the reaction mixture was refluxed for 12 h. After cooling to room temperature, the mixture was poured into water and extracted twice with dichloromethane, and then dried over anhydrous magnesium sulfate. After filtration, the solvent was evaporated under reduced pressure and the residue was purified by silica-gel column chromatography (dichloromethane/petroleum). White solid of BICZ was obtained in 76% yield (1.35 g). ^1^H NMR (400 MHz, CD_2_Cl_2_), δ (TMS, ppm): δ 8.26 (dd, J = 10.9, 8.0 Hz, 2H), 8.17 (d, J = 1.6 Hz, 2H), 7.99 (d, J = 8.6 Hz, 2H), 7.91 – 7.82 (m, 5H), 7.74 (s, 1H), 7.73 – 7.68 (m, 3H), 7.68 – 7.61 (m, 6H), 7.60 – 7.53 (m, 3H), 7.49 (dd, J = 8.7, 1.9 Hz, 2H), 7.43 – 7.24 (m, 7H), 1.47 (s, 18H). ^13^C NMR (126 MHz, CD_2_Cl_2_) δ 152.13, 143.48, 142.76, 142.04, 141.85, 141.74, 140.36, 139.56, 139.53, 139.07, 137.84, 137.41, 134.71, 130.41, 130.31, 129.19, 129.10, 128.03, 127.96, 127.54, 127.26, 124.13, 123.84, 123.79, 123.67, 123.46, 123.36, 121.45, 121.39, 121.00, 120.89, 119.80, 118.68, 116.73, 111.49, 110.93, 109.60, 108.48, 108.40, 35.05, 32.15. HRMS (C_64_H_51_N_5_): m/z 889.4144 (M + H^+^, calcd 890.4269).

**(3,5-di(pyridin-3-yl)phenyl)boronic acid:** The compound 3,3 '-(5-bromo-1, 3-phenyl) dipyridine (2.4 mmol, 746.8 mg) was dissolved in 100ml dry tetrahydrofuran in a nitrogen atmosphere. After stirring at -78 ^o^C for 0.5 hours, n-butyllithium (2.4 mmol) was slowly added, and the reaction was maintained at -78K for 3 hours. Then slowly add trimethyl borate (4.8 mmol, 498.7mg), after 2 hours, slowly heat to room temperature overnight. After quenching reaction with water, the white solid of (3,5-di(pyridin-3-yl)phenyl)boronic acid was extracted and filtered. The product is directly used for subsequent reactions without further purification.

**4-(2-(3,5-di(pyridin-3-yl)phenyl)-7-(4-(3,6-di-tert-butyl-9H-carbazol-9-yl)phenyl)-9H-carbazol-9-yl)benzonitrile(PHDPYCZ):** A mixture of compound Br-CNCz-BuCz (2.1 g, 3 mmol), (3,5-di(pyridin-3-yl)phenyl)boronic acid (0.994 g, 3.6 mmol), Pd(PPh3)4 (0.346 g, 0.3 mmol), and potassium carbonate (1.24 g, 9 mmol) was added in 250 mL two-neck bottle under nitrogen. Then, a mixed solvent system of toluene, C_2_H_5_OH and H_2_O (v/v/v = 8:1:1) was injected into the bottle, and the reaction mixture was refluxed for 12 h. After cooling to room temperature, the mixture was poured into water and extracted twice with dichloromethane, and then dried over anhydrous magnesium sulfate. After filtration, the solvent was evaporated under reduced pressure and the residue was purified by silica-gel column chromatography (dichloromethane/petroleum). White solid of PHDPYCZ was obtained in 84% yield (2.14 g). ^1^H NMR (500 MHz, CD_2_Cl_2_), δ (TMS, ppm): δ 8.99 (d, J = 2.0 Hz, 2H), 8.67 – 8.64 (m, 2H), 8.32 (dd, J = 7.9, 5.3 Hz, 2H), 8.18 (d, J = 1.6 Hz, 2H), 8.10 (d, J = 7.9 Hz, 2H), 7.99 (d, J = 8.4 Hz, 2H), 7.95 – 7.92 (m, 2H), 7.89 (t, J = 8.3 Hz, 4H), 7.82 (s, 1H), 7.77 – 7.72 (m, 4H), 7.67 (d, J = 8.4 Hz, 2H), 7.53 – 7.48 (m, 4H), 7.42 (d, J = 8.6 Hz, 2H), 1.47 (s, 18H). ^13^C NMR (126 MHz, CD_2_Cl_2_) δ 149.58, 149.03, 144.12, 143.76, 142.28, 142.16, 142.03, 140.62, 140.18, 139.90, 139.87, 139.80, 138.14, 136.86, 135.25, 135.04, 129.39, 128.31, 127.54, 126.79, 125.74, 124.40, 124.37, 124.07, 124.03, 123.60, 121.77, 121.52, 121.31, 118.94, 117.02, 111.86, 109.87, 109.03, 108.81, 35.32, 32.43. HRMS (C_61_H_49_N_5_): m/z 851.3988 (M + H^+^, calcd 852.4063).

**4-(2-(4-(3,6-di-tert-butyl-9H-carbazol-9-yl)phenyl)-7-(4-(pyridin-3-yl)phenyl)-9H-carbazol-9-yl)benzonitrile(PHPYCZ):** A mixture of compound Br-CNCz-BuCz (2.1 g, 3 mmol), (4-(pyridin-3-yl)phenyl)boronic acid (0.716 g, 3.6 mmol), Pd(PPh3)4 (0.346 g, 0.3 mmol), and potassium carbonate (1.24 g, 9 mmol) was added in 250 mL two-neck bottle under nitrogen. Then, a mixed solvent system of toluene, C_2_H_5_OH and H_2_O (v/v/v = 8:1:1) was injected into the bottle, and the reaction mixture was refluxed for 12 h. After cooling to room temperature, the mixture was poured into water and extracted twice with dichloromethane, and then dried over anhydrous magnesium sulfate. After filtration, the solvent was evaporated under reduced pressure and the residue was purified by silica-gel column chromatography (dichloromethane/petroleum). White solid of PHPYCZ was obtained in 80% yield (1.86 g). ^1^H NMR (400 MHz, CD_2_Cl_2_), δ (TMS, ppm): δ 8.92 (d, J = 1.9 Hz, 1H), 8.63 – 8.56 (m, 1H), 8.29 (dd, J = 8.1, 5.2 Hz, 2H), 8.18 (d, J = 1.5 Hz, 2H), 8.06 – 7.97 (m, 3H), 7.89 (dd, J = 8.5, 4.8 Hz, 4H), 7.82 (d, J = 8.4 Hz, 2H), 7.77 – 7.64 (m, 8H), 7.52 – 7.40 (m, 5H), 1.47 (s, 18H). ^13^C NMR (126 MHz, CD_2_Cl_2_) δ 149.58, 149.03, 144.12, 143.76, 142.28, 142.16, 142.03, 140.62, 140.18, 139.90, 139.87, 139.80, 138.14, 136.86, 135.25, 135.04, 129.39, 128.31, 127.54, 126.79, 125.74, 124.40, 124.37, 124.07, 124.03, 123.60, 121.77, 121.52, 121.31, 118.94, 117.02, 111.86, 109.87, 109.03, 108.81, 35.32, 32.43. HRMS (C_56_H_46_N_4_): m/z 774.3722 (M + H^+^, calcd 775.3818). 775.3795

- 1. **Thermodynamic and electrochemical analysis**


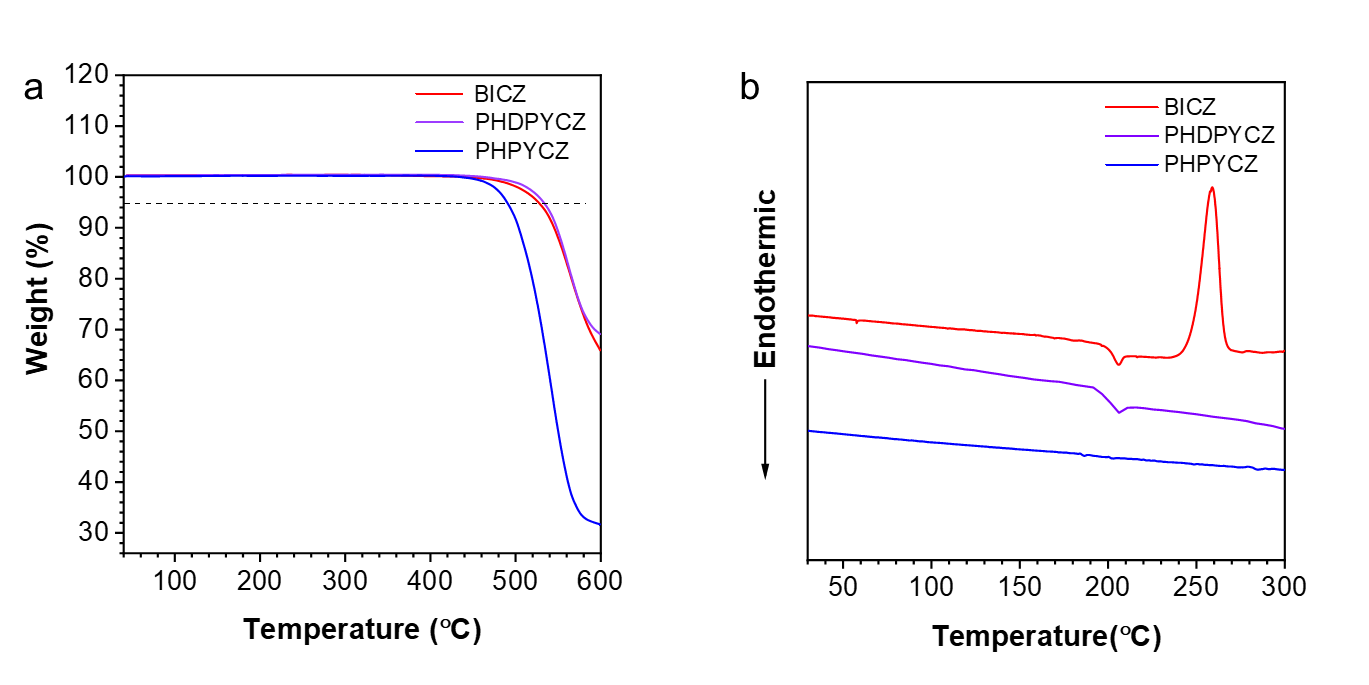


**Supplementary Figure 1** (a) Thermogravimetric analysis and (b) differential scanning calorimetry curve of **BICZ**, **PHDPYCZ** and **PHPYCZ**.


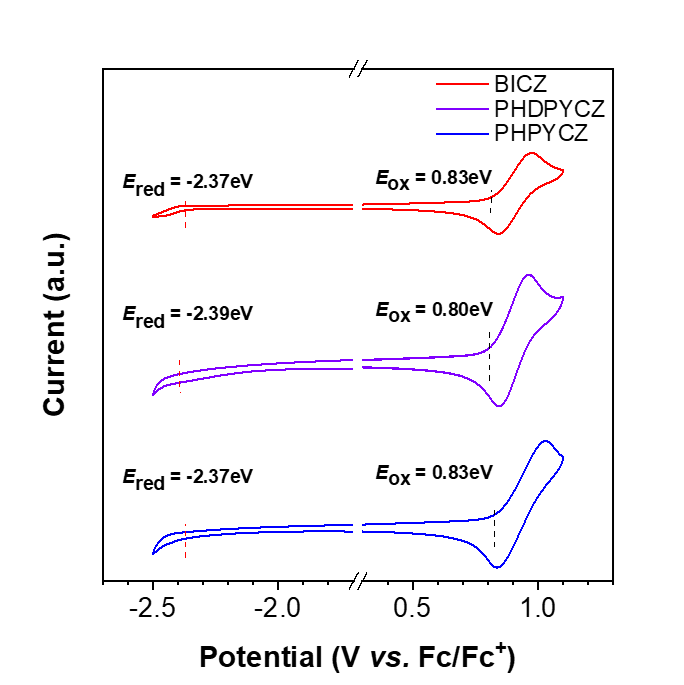


**Supplementary Figure 2** Cyclic voltammogram of the investigated target molecules.

1. **Single crystal data**

CCDC 2362108 contains the supplementary crystallographic data for this paper. These data can be obtained free of charge from The Cambridge Crystallographic Data Centre via www.ccdc.cam.ac.uk/data_request/cif.

**Supplementary Table 1** Crystal data and structure refinement for **PHPYCZ**.

| Empirical formula | C_56_H_46_N_4_ |
| --- | --- |
| Formula weight | 774.97 |
| Temperature/K | 293(2) |
| Crystal system | triclinic |
| Space group | P-1 |
| a/Å | 6.7096(2) |
| b/Å | 16.8950(4) |
| c/Å | 20.1324(5) |
| α/° | 111.274(2) |
| β/° | 90.843(2) |
| γ/° | 91.569(2) |
| Volume/Å^3^ | 2125.12(10) |
| Z | 2 |
| ρ_calc_g/cm^3^ | 0.542 |
| μ/mm^-1^ | 2.086 |
| F(000) | 820 |
| Crystal size/mm^3^ | 0.5 × 0.5 × 0.2 |
| Radiation | Cu Kα (profit = 1.54184) |
| 2Θ range for data collection/° | 4.712 to 153.31 |
| Index ranges | -8 ≤ h ≤ 8, -21 ≤ k ≤ 19, -24 ≤ l ≤ 25 |
| Reflections collected | 29667 |
| Independent reflections | 8614 [R_int_ = 0.0251, R_sigma_ = 0.0246] |
| Data/restraints/parameters | 8614/1/584 |
| Goodness-of-fit on F^2^ | 1.056 |
| Final R indexes [I>=2σ (I)] | R_1_ = 0.0709, wR_2_ = 0.2138 |
| Final R indexes [all data] | R_1_ = 0.0788, wR_2_ = 0.2236 |
| Largest diff. peak/hole / e Å^-3^ | 0.59/-0.34 |


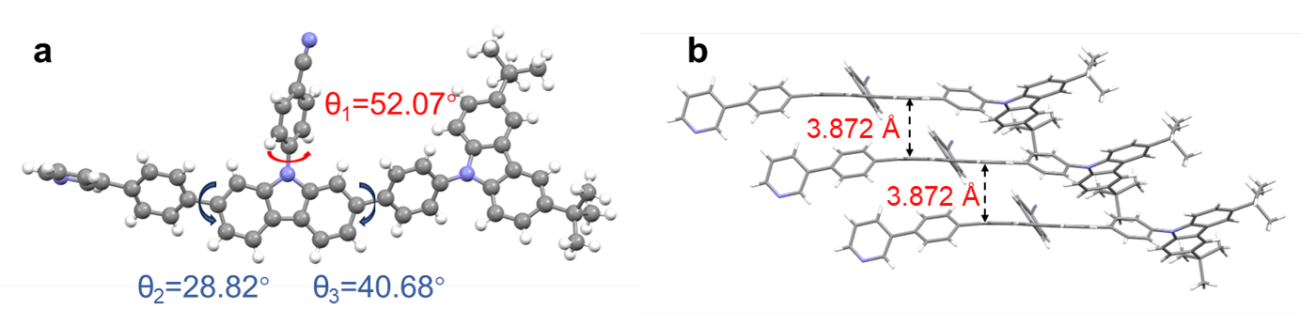


**Supplementary Figure 3** Crystal structure and packing pattern of PHPYCZ in crystals

1. **Computational Methods**

All density functional theory (DFT) and time-dependent DFT (TD-DFT) calculations were carried out using Gaussian 16 package. The optimized structure were calculated by DFT method at the M062X/6–31G (d, p) level. The S_1_ geometry was optimized by time-dependent DFT (TD-DFT) at the TD-M062X /6–31G (d, p) level. For better understanding of the excited-state properties, natural transition orbitals (NTO) and energy levels of the first five singlet and triplet states were performed on the basis of S_1_ geometry at the same level.

**Supplementary Table 2** Cartesian coordinates for the optimized structure of ground-state (S_0_) geometry of BICZ.

| Atom | x | y | z |
| --- | --- | --- | --- |
| C | 2.6457945 | -1.07301589 | -0.44233067 |
| C | 2.22847993 | -2.41852162 | -0.51481656 |
| C | 0.78469451 | -2.4170203 | -0.42286732 |
| C | 0.38315375 | -1.07091559 | -0.29479383 |
| N | 1.51853789 | -0.25791039 | -0.31186261 |
| C | -0.18727901 | -3.41849778 | -0.45748021 |
| C | -1.52396626 | -3.06530566 | -0.37498135 |
| C | -1.921931 | -1.71632488 | -0.27091856 |
| C | -0.95969556 | -0.70593023 | -0.23442231 |
| C | 3.99228593 | -0.71814545 | -0.4645954 |
| C | 4.94198376 | -1.73343453 | -0.59065842 |
| C | 4.52807027 | -3.07845786 | -0.68255161 |
| C | 3.18789061 | -3.42500967 | -0.63934574 |
| C | 0.80646456 | 3.15086178 | 0.93189195 |
| C | 0.80603611 | 1.7684179 | 0.82452639 |
| C | 1.52554425 | 1.14630421 | -0.20064889 |
| C | 2.2518305 | 1.91647416 | -1.11414655 |
| C | 2.26841887 | 3.29833863 | -0.99694728 |
| C | 1.54223139 | 3.92033991 | 0.02420394 |
| C | -8.51315757 | -1.25386632 | 0.15244485 |
| C | -9.72918164 | -0.53883545 | 0.15193239 |
| C | -9.39227031 | 0.85785858 | -0.04794424 |
| C | -7.9875702 | 0.92488724 | -0.15740337 |
| N | -7.45816562 | -0.36031072 | -0.03490389 |
| C | -10.15223446 | 2.01965582 | -0.15500447 |
| C | -9.53486294 | 3.25182316 | -0.37743981 |
| C | -8.13379781 | 3.27902433 | -0.50027224 |
| C | -7.34693251 | 2.13686282 | -0.39779071 |
| C | -8.49364775 | -2.62949891 | 0.36316074 |
| C | -9.71113141 | -3.27636538 | 0.54654079 |
| C | -10.94334587 | -2.59804454 | 0.53322482 |
| C | -10.93153654 | -1.21566552 | 0.33855908 |
| C | -10.39568092 | 4.51568039 | -0.48707261 |
| C | -12.2820461 | -3.31855548 | 0.73074292 |
| C | -12.10503599 | -4.82763156 | 0.92840536 |
| C | -12.99052056 | -2.75178272 | 1.97264841 |
| C | -9.55320142 | 5.77219844 | -0.73026979 |
| C | -11.38079707 | 4.3641913 | -1.65844608 |
| C | -11.18442405 | 4.71190592 | 0.81860311 |
| C | -6.08925128 | -0.69782721 | -0.09131314 |
| C | -5.16093787 | -0.0000483 | 0.68460925 |
| C | -3.81391332 | -0.32877419 | 0.61697413 |
| C | -3.36135322 | -1.36750118 | -0.20540641 |
| C | -4.30584712 | -2.06502096 | -0.96820036 |
| C | -5.65271916 | -1.7313626 | -0.92322845 |
| C | -13.16902294 | -3.09741241 | -0.50610173 |
| C | 14.9000918 | 0.76934723 | -0.59944703 |
| C | 14.82206519 | 0.36046545 | -1.94470381 |
| C | 13.63778604 | -0.10857407 | -2.48840038 |
| C | 12.5114574 | -0.15992661 | -1.65971895 |
| C | 12.60463932 | 0.27015684 | -0.3233655 |
| C | 13.79569708 | 0.729973 | 0.23861939 |
| N | 11.2305192 | -0.59304707 | -1.9357419 |
| C | 10.56046571 | -0.43907345 | -0.82204602 |
| N | 11.3351059 | 0.08401205 | 0.20559091 |
| C | 9.12685231 | -0.7544529 | -0.70138711 |
| C | 11.0091443 | 0.23303079 | 1.58170245 |
| C | 11.19772889 | 1.47035591 | 2.19549221 |
| C | 10.8935622 | 1.61954793 | 3.54561981 |
| C | 10.39086763 | 0.54388584 | 4.27277821 |
| C | 10.20193415 | -0.68860696 | 3.65054493 |
| C | 10.51984596 | -0.85251708 | 2.30706139 |
| C | 8.25664456 | -0.05103615 | 0.13831242 |
| C | 6.90624079 | -0.37295726 | 0.17336278 |
| C | 6.38554853 | -1.39572879 | -0.62770265 |
| C | 7.26158463 | -2.08524696 | -1.47596136 |
| C | 8.61104017 | -1.7689 | -1.51545005 |
| C | 1.55172041 | 5.35404697 | 0.14254464 |
| N | 1.55900483 | 6.50715547 | 0.23976839 |
| H | 0.1013736 | -4.461417 | -0.54391115 |
| H | -2.28529159 | -3.83835296 | -0.3686966 |
| H | -1.25992152 | 0.33612024 | -0.20333 |
| H | 4.3044374 | 0.31890396 | -0.4058639 |
| H | 5.28248071 | -3.8548394 | -0.75485979 |
| H | 2.88769784 | -4.46707221 | -0.68866907 |
| H | 0.25369393 | 3.64099089 | 1.72549364 |
| H | 0.26486272 | 1.15835217 | 1.53988629 |
| H | 2.78542673 | 1.42422121 | -1.92000102 |
| H | 2.82815719 | 3.90243368 | -1.70211828 |
| H | -11.23407077 | 1.95601066 | -0.06855741 |
| H | -7.6309766 | 4.22090553 | -0.68618406 |
| H | -6.27027986 | 2.19423315 | -0.51383782 |
| H | -7.5620573 | -3.18364748 | 0.39494172 |
| H | -9.69162895 | -4.34783293 | 0.70829609 |
| H | -11.86250839 | -0.65423503 | 0.33550229 |
| H | -13.084889 | -5.29595378 | 1.0608503 |
| H | -11.62624922 | -5.29366472 | 0.06149037 |
| H | -11.5058351 | -5.05009996 | 1.81686186 |
| H | -13.94996542 | -3.25734071 | 2.12596465 |
| H | -13.18788229 | -1.68113979 | 1.86940424 |
| H | -12.37705823 | -2.89630671 | 2.86693173 |
| H | -10.20992722 | 6.64461767 | -0.79763074 |
| H | -8.84687092 | 5.94796535 | 0.08711529 |
| H | -8.99040575 | 5.70496313 | -1.66653956 |
| H | -12.00387376 | 5.26044077 | -1.74852893 |
| H | -12.04448642 | 3.50658213 | -1.51718276 |
| H | -10.8423525 | 4.22183554 | -2.60013197 |
| H | -10.50403028 | 4.82204349 | 1.66828989 |
| H | -11.80617943 | 5.61133607 | 0.75493657 |
| H | -11.84232023 | 3.86236804 | 1.02221291 |
| H | -5.50834897 | 0.78091651 | 1.35321659 |
| H | -3.10358608 | 0.20213958 | 1.24378056 |
| H | -3.97348693 | -2.85183869 | -1.63839684 |
| H | -6.37303719 | -2.25008748 | -1.54748269 |
| H | -12.68546464 | -3.49327632 | -1.40415426 |
| H | -14.13108687 | -3.60532629 | -0.37923688 |
| H | -13.36916228 | -2.03522654 | -0.6723229 |
| H | 15.84825503 | 1.12285461 | -0.20806814 |
| H | 15.71220779 | 0.41173774 | -2.5627205 |
| H | 13.56827998 | -0.43456049 | -3.52022608 |
| H | 13.85557142 | 1.03554053 | 1.27770835 |
| H | 11.56979077 | 2.30312581 | 1.60684809 |
| H | 11.04048541 | 2.58139296 | 4.02543119 |
| H | 10.14743202 | 0.66466507 | 5.32295865 |
| H | 9.81388128 | -1.52967879 | 4.21520381 |
| H | 10.38591199 | -1.80757027 | 1.80977289 |
| H | 8.62865807 | 0.75473513 | 0.76197968 |
| H | 6.24731875 | 0.16520643 | 0.84794141 |
| H | 6.87230338 | -2.85937618 | -2.130139 |
| H | 9.28696663 | -2.28614202 | -2.18773757 |

**Supplementary Table 3** Cartesian coordinates for the optimized structure of ground-state (S_0_) geometry of PHDPYCZ.

| Atom | x | y | z |
| --- | --- | --- | --- |
| C | -3.60016225 | -0.76024231 | -0.07916506 |
| C | -3.23759319 | -2.11886557 | -0.19423375 |
| C | -1.79208679 | -2.16809651 | -0.21792092 |
| C | -1.33455076 | -0.83788788 | -0.11754535 |
| N | -2.43839122 | 0.01342427 | -0.02820408 |
| C | -0.86184056 | -3.20597322 | -0.29777647 |
| C | 0.48938136 | -2.90457999 | -0.26752071 |
| C | 0.94298327 | -1.57492671 | -0.14206436 |
| C | 0.0225379 | -0.52880896 | -0.0607881 |
| C | -4.93230207 | -0.35337584 | -0.07091552 |
| C | -5.92233946 | -1.33370758 | -0.15096891 |
| C | -5.56501613 | -2.69421554 | -0.24340137 |
| C | -4.23779934 | -3.089358 | -0.27193619 |
| C | -1.53020907 | 3.53615925 | -0.69176583 |
| C | -1.58943411 | 2.15515085 | -0.80151616 |
| C | -2.38683669 | 1.41731728 | 0.07897982 |
| C | -3.13200685 | 2.07101399 | 1.06504607 |
| C | -3.08867514 | 3.45375016 | 1.16399125 |
| C | -2.28372382 | 4.19051744 | 0.28872725 |
| C | 7.55525144 | -1.36810078 | -0.27033645 |
| C | 8.8017075 | -0.72566802 | -0.117269 |
| C | 8.52182618 | 0.63288636 | 0.30735001 |
| C | 7.11863386 | 0.75190656 | 0.38879138 |
| N | 6.53606484 | -0.46646171 | 0.03811756 |
| C | 9.32962192 | 1.72048456 | 0.6288986 |
| C | 8.76146489 | 2.92814672 | 1.03831969 |
| C | 7.35961223 | 3.00585896 | 1.12508885 |
| C | 6.52560883 | 1.93809462 | 0.81102615 |
| C | 7.48011362 | -2.68562444 | -0.712461 |
| C | 8.6722619 | -3.35254397 | -0.97379261 |
| C | 9.93288583 | -2.74901001 | -0.8148876 |
| C | 9.97753575 | -1.42106368 | -0.38661469 |
| C | 9.67420415 | 4.11080017 | 1.38260435 |
| C | 11.24324687 | -3.49181144 | -1.10046021 |
| C | 11.00437361 | -4.9333019 | -1.56179813 |
| C | 12.01854906 | -2.75640407 | -2.20658067 |
| C | 8.88131322 | 5.35039295 | 1.80983841 |
| C | 10.60869005 | 3.71679311 | 2.53887322 |
| C | 10.51741645 | 4.48141107 | 0.15089687 |
| C | 5.15236835 | -0.73899745 | -0.00440269 |
| C | 4.28670081 | 0.12117735 | -0.68364897 |
| C | 2.92557557 | -0.14949494 | -0.71747618 |
| C | 2.39642257 | -1.28636083 | -0.09479225 |
| C | 3.27852282 | -2.14319153 | 0.57478297 |
| C | 4.63889801 | -1.87215119 | 0.63050911 |
| C | 12.09695143 | -3.53239094 | 0.17823709 |
| C | -10.62817452 | -2.14433876 | 1.33686362 |
| C | -11.67569141 | -1.55916319 | 2.05170973 |
| C | -9.64250654 | -1.33265188 | 0.5861139 |
| C | -10.04888387 | -0.19178643 | -0.11100571 |
| C | -8.29390769 | -1.69712038 | 0.56281146 |
| C | -7.3543583 | -0.94246669 | -0.14546523 |
| C | -7.78495818 | 0.19535212 | -0.83236306 |
| C | -9.12755871 | 0.58153278 | -0.8215345 |
| C | -10.54951565 | -3.54260607 | 1.35163989 |
| N | -11.39388913 | -4.33638428 | 2.00624113 |
| C | -12.3826157 | -3.7525036 | 2.68593843 |
| C | -12.56867934 | -2.37339055 | 2.73511563 |
| C | -9.57016711 | 1.78135089 | -1.56923051 |
| C | -10.5346508 | 2.64630538 | -1.03739272 |
| N | -10.98439648 | 3.73913145 | -1.64986162 |
| C | -10.47208655 | 4.02529698 | -2.8482008 |
| C | -9.50159971 | 3.24590692 | -3.47254688 |
| C | -9.04707376 | 2.10624541 | -2.82286306 |
| C | -2.22941803 | 5.62409697 | 0.39644073 |
| N | -2.18450704 | 6.77725939 | 0.48176492 |
| H | -1.19366654 | -4.2356074 | -0.38817321 |
| H | 1.22000539 | -3.70088294 | -0.36354548 |
| H | 0.36291368 | 0.49103789 | 0.08398682 |
| H | -5.20329404 | 0.69367103 | 0.01256643 |
| H | -6.35053604 | -3.43798765 | -0.32809595 |
| H | -3.97873772 | -4.13915698 | -0.36743471 |
| H | -0.9161459 | 4.11604145 | -1.37164277 |
| H | -1.03403975 | 1.63759015 | -1.57641018 |
| H | -3.7271666 | 1.48617001 | 1.7580267 |
| H | -3.66228542 | 3.96813755 | 1.92679679 |
| H | 10.40980863 | 1.61729046 | 0.562172 |
| H | 6.8939135 | 3.92822279 | 1.45200635 |
| H | 5.44919955 | 2.03029749 | 0.90500943 |
| H | 6.52581346 | -3.17885127 | -0.86088403 |
| H | 8.60947518 | -4.37871052 | -1.31671003 |
| H | 10.93247282 | -0.91600831 | -0.26459722 |
| H | 11.96579538 | -5.42046665 | -1.7496554 |
| H | 10.47681427 | -5.51727868 | -0.80113701 |
| H | 10.42516689 | -4.96861238 | -2.48979631 |
| H | 12.95835087 | -3.27631763 | -2.42122792 |
| H | 12.2614447 | -1.73107867 | -1.91399288 |
| H | 11.42968607 | -2.71301947 | -3.12763781 |
| H | 9.57402015 | 6.16516536 | 2.04055 |
| H | 8.2132707 | 5.69504402 | 1.01433028 |
| H | 8.28306664 | 5.15717652 | 2.70572748 |
| H | 11.26792817 | 4.55290711 | 2.79560684 |
| H | 11.23766712 | 2.86225346 | 2.27420988 |
| H | 10.03086448 | 3.44694357 | 3.42783497 |
| H | 9.87370355 | 4.76606719 | -0.68674171 |
| H | 11.17687105 | 5.32479569 | 0.38205892 |
| H | 11.14278982 | 3.64531726 | -0.17443016 |
| H | 4.69390327 | 0.98353714 | -1.20149973 |
| H | 2.2651025 | 0.51005576 | -1.27251305 |
| H | 2.88653049 | -3.01210607 | 1.09437222 |
| H | 5.31085631 | -2.52068189 | 1.18325576 |
| H | 11.56545617 | -4.05168285 | 0.9812856 |
| H | 13.03841644 | -4.05937197 | -0.01033622 |
| H | 12.33985225 | -2.52626852 | 0.53131486 |
| H | -11.7721345 | -0.47760419 | 2.08626595 |
| H | -11.10312211 | 0.06873859 | -0.14328158 |
| H | -7.96116362 | -2.55244175 | 1.14376179 |
| H | -7.0629633 | 0.78861538 | -1.38681821 |
| H | -9.76666179 | -4.04134973 | 0.78262387 |
| H | -13.05874547 | -4.41893542 | 3.21591632 |
| H | -13.38861213 | -1.95128016 | 3.30531334 |
| H | -10.94780906 | 2.44590547 | -0.05011623 |
| H | -10.85541175 | 4.92027018 | -3.3321256 |
| H | -9.12204394 | 3.52305104 | -4.44965175 |
| H | -8.31021127 | 1.45800673 | -3.28865997 |

**Supplementary Table 4** Cartesian coordinates for the optimized structure of ground-state (S_0_) geometry of PHPYCZ.

| Atom | x | y | z |
| --- | --- | --- | --- |
| C | -4.42789511 | -0.93008017 | 0.05951131 |
| C | -4.03625298 | -2.27922123 | 0.18813961 |
| C | -2.59026361 | -2.30303203 | 0.15422852 |
| C | -2.16145125 | -0.96786871 | 0.0034056 |
| N | -3.28293055 | -0.13709635 | -0.04940628 |
| C | -1.63756095 | -3.3183146 | 0.25702961 |
| C | -0.29301585 | -2.98862746 | 0.21894237 |
| C | 0.13155895 | -1.64961448 | 0.09290966 |
| C | -0.81125869 | -0.62579512 | -0.0124361 |
| C | -5.76839455 | -0.5542272 | 0.01782619 |
| C | -6.73857267 | -1.5508552 | 0.13567101 |
| C | -6.35102363 | -2.89867992 | 0.28321111 |
| C | -5.01608971 | -3.26679808 | 0.30375162 |
| C | -2.46140324 | 3.22233206 | -1.35839825 |
| C | -2.49118199 | 1.84360362 | -1.21344034 |
| C | -3.26115574 | 1.26337422 | -0.20050249 |
| C | -4.00831663 | 2.0714502 | 0.6618956 |
| C | -3.99429762 | 3.44959112 | 0.50653873 |
| C | -3.21699548 | 4.02982551 | -0.5014592 |
| C | 6.7411178 | -1.31069297 | -0.06557152 |
| C | 7.96928988 | -0.61768525 | -0.02970533 |
| C | 7.6506131 | 0.78938653 | 0.12084274 |
| C | 6.24402097 | 0.88443492 | 0.1667895 |
| N | 5.69617742 | -0.39387527 | 0.05346286 |
| C | 8.42732608 | 1.93961943 | 0.23222546 |
| C | 7.8245254 | 3.18802578 | 0.39615334 |
| C | 6.42019323 | 3.24367103 | 0.45601503 |
| C | 5.61676686 | 2.11360989 | 0.34785093 |
| C | 6.70461267 | -2.69065415 | -0.24311054 |
| C | 7.91641486 | -3.36350464 | -0.35821078 |
| C | 9.15950958 | -2.70704718 | -0.30852937 |
| C | 9.16555448 | -1.32029648 | -0.14780139 |
| C | 8.70372895 | 4.43859003 | 0.5121704 |
| C | 10.4915128 | -3.45587756 | -0.43193816 |
| C | 10.29463532 | -4.96588753 | -0.60171055 |
| C | 11.2620316 | -2.93196059 | -1.65557421 |
| C | 7.87554121 | 5.7160684 | 0.68507941 |
| C | 9.63250509 | 4.30014558 | 1.73024582 |
| C | 9.55354724 | 4.58616117 | -0.76118835 |
| C | 4.32015422 | -0.70593765 | 0.06111909 |
| C | 3.43754333 | -0.01383479 | -0.77113765 |
| C | 2.08314743 | -0.31795215 | -0.75153653 |
| C | 1.57832067 | -1.32614262 | 0.07832168 |
| C | 2.47764674 | -2.01832772 | 0.89842187 |
| C | 3.83096525 | -1.70894589 | 0.90105477 |
| C | 11.32974131 | -3.22074966 | 0.83593002 |
| C | -10.91629497 | -0.49891328 | 0.05062593 |
| C | -9.9967724 | 0.14743809 | -0.78324375 |
| C | -8.65081086 | -0.19234644 | -0.75656758 |
| C | -8.17669836 | -1.1901782 | 0.10292043 |
| C | -9.09793827 | -1.83771424 | 0.93467828 |
| C | -10.44341043 | -1.49726585 | 0.90966856 |
| C | -12.35113246 | -0.13774036 | 0.02511446 |
| C | -12.75897969 | 1.19443681 | -0.12240421 |
| N | -14.02850988 | 1.59314512 | -0.15881667 |
| C | -14.97006226 | 0.65523692 | -0.03923717 |
| C | -14.68643923 | -0.69827114 | 0.12316222 |
| C | -13.35738861 | -1.09838355 | 0.15277435 |
| C | -3.1935899 | 5.45976388 | -0.65755803 |
| N | -3.1739694 | 6.60986655 | -0.78403627 |
| H | -1.94702994 | -4.3535962 | 0.36139941 |
| H | 0.45477195 | -3.7733782 | 0.26591414 |
| H | -0.49263208 | 0.41009179 | -0.06138998 |
| H | -6.06131088 | 0.48512916 | -0.08477097 |
| H | -7.12049116 | -3.66102234 | 0.34627375 |
| H | -4.73505823 | -4.31140809 | 0.39469421 |
| H | -1.86908284 | 3.68008577 | -2.14273541 |
| H | -1.93414473 | 1.20424752 | -1.88997353 |
| H | -4.58199952 | 1.61138958 | 1.45919753 |
| H | -4.56958554 | 4.08321031 | 1.17217908 |
| H | 9.51048946 | 1.85416867 | 0.19521094 |
| H | 5.9278845 | 4.19905893 | 0.59533482 |
| H | 4.53735664 | 2.19348316 | 0.41445356 |
| H | 5.76486018 | -3.22865046 | -0.3015168 |
| H | 7.88372393 | -4.43816848 | -0.49470807 |
| H | 10.10603231 | -0.77572749 | -0.11818511 |
| H | 11.27021951 | -5.45451894 | -0.68201151 |
| H | 9.77161965 | -5.40255982 | 0.25486858 |
| H | 9.72884493 | -5.1989173 | -1.50915974 |
| H | 12.21717447 | -3.45841075 | -1.75658727 |
| H | 11.47545111 | -1.86293316 | -1.56890903 |
| H | 10.68381354 | -3.08660652 | -2.57138834 |
| H | 8.54524539 | 6.57800426 | 0.75929252 |
| H | 7.20955815 | 5.88277579 | -0.16732539 |
| H | 7.2707195 | 5.68383769 | 1.59666595 |
| H | 10.26829488 | 5.18689471 | 1.82534882 |
| H | 10.28491311 | 3.42701655 | 1.64112504 |
| H | 9.04992237 | 4.192659 | 2.65003626 |
| H | 8.91403749 | 4.68572783 | -1.64330759 |
| H | 10.18865339 | 5.4758424 | -0.69262745 |
| H | 10.20382294 | 3.72009533 | -0.91307965 |
| H | 3.82639392 | 0.74275785 | -1.44493155 |
| H | 1.40923919 | 0.20745279 | -1.42169624 |
| H | 2.10383276 | -2.78096806 | 1.57469111 |
| H | 4.51544386 | -2.2227709 | 1.56825249 |
| H | 10.80165269 | -3.58629235 | 1.72167042 |
| H | 12.28667539 | -3.74841257 | 0.76168568 |
| H | 11.54250607 | -2.15856441 | 0.98569628 |
| H | -10.34690628 | 0.89923169 | -1.4842067 |
| H | -7.96088057 | 0.29672537 | -1.43782233 |
| H | -8.74762294 | -2.5917227 | 1.63309047 |
| H | -11.1338001 | -1.9877049 | 1.5898062 |
| H | -12.00840261 | 1.97964517 | -0.19523883 |
| H | -15.99986823 | 1.00233968 | -0.0746681 |
| H | -15.49110499 | -1.41962146 | 0.21227411 |
| H | -13.09587318 | -2.14824264 | 0.25087022 |
| H | -13.09587318 | -2.14824264 | 0.25087022 |


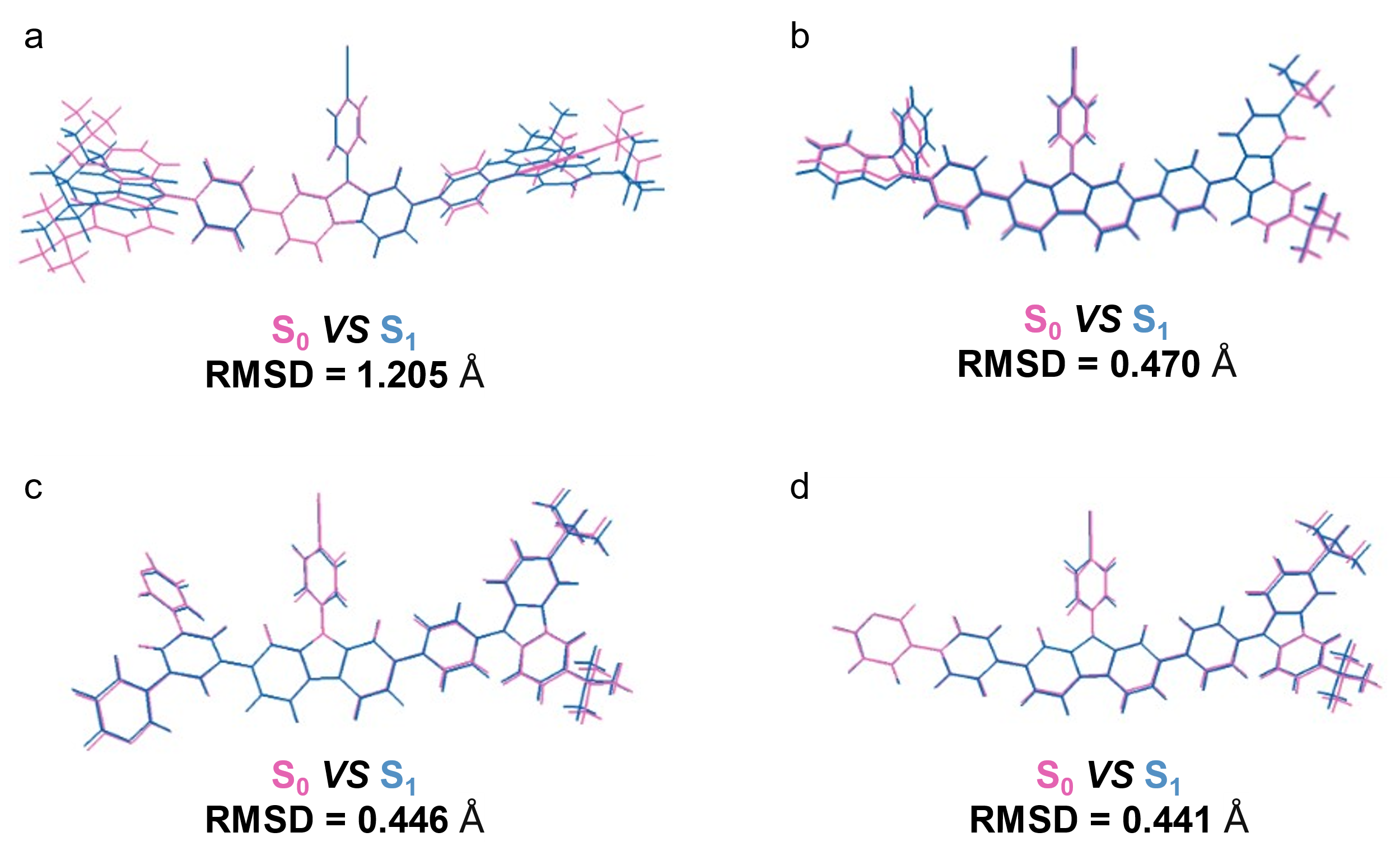


**Supplementary Figure 4** Theoretical calculation RMSD results a) 2BuCz-CNCz, b) BICZ, c) PHDPYCZ, and d) PHPYCZ.


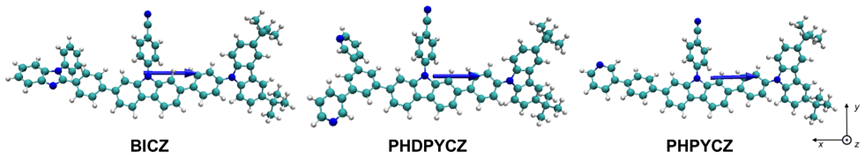


**Supplementary Figure 5** Transition dipole moment of S_1_ state is indicated by blue arrow. The transition dipole moments of BICZ, PHDPYCZ, and PHPYCZ are calculated to be preferentially oriented along the long-axis skeleton (in the x-y plane).


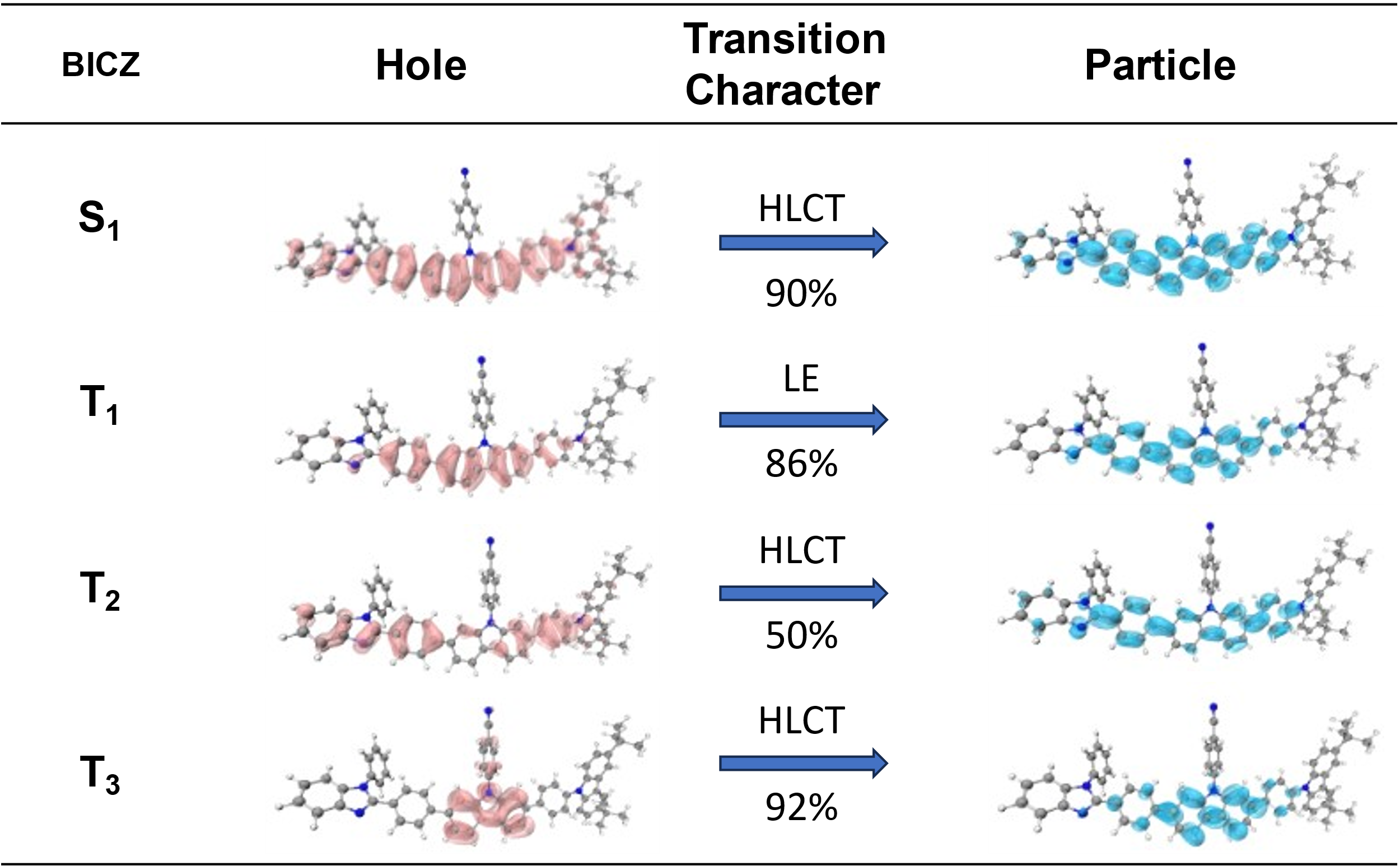


**Supplementary Figure 6** NTOs describing the transition characters of the S_1_, T_1_, T_2_ and T_3_ states in BICZ.


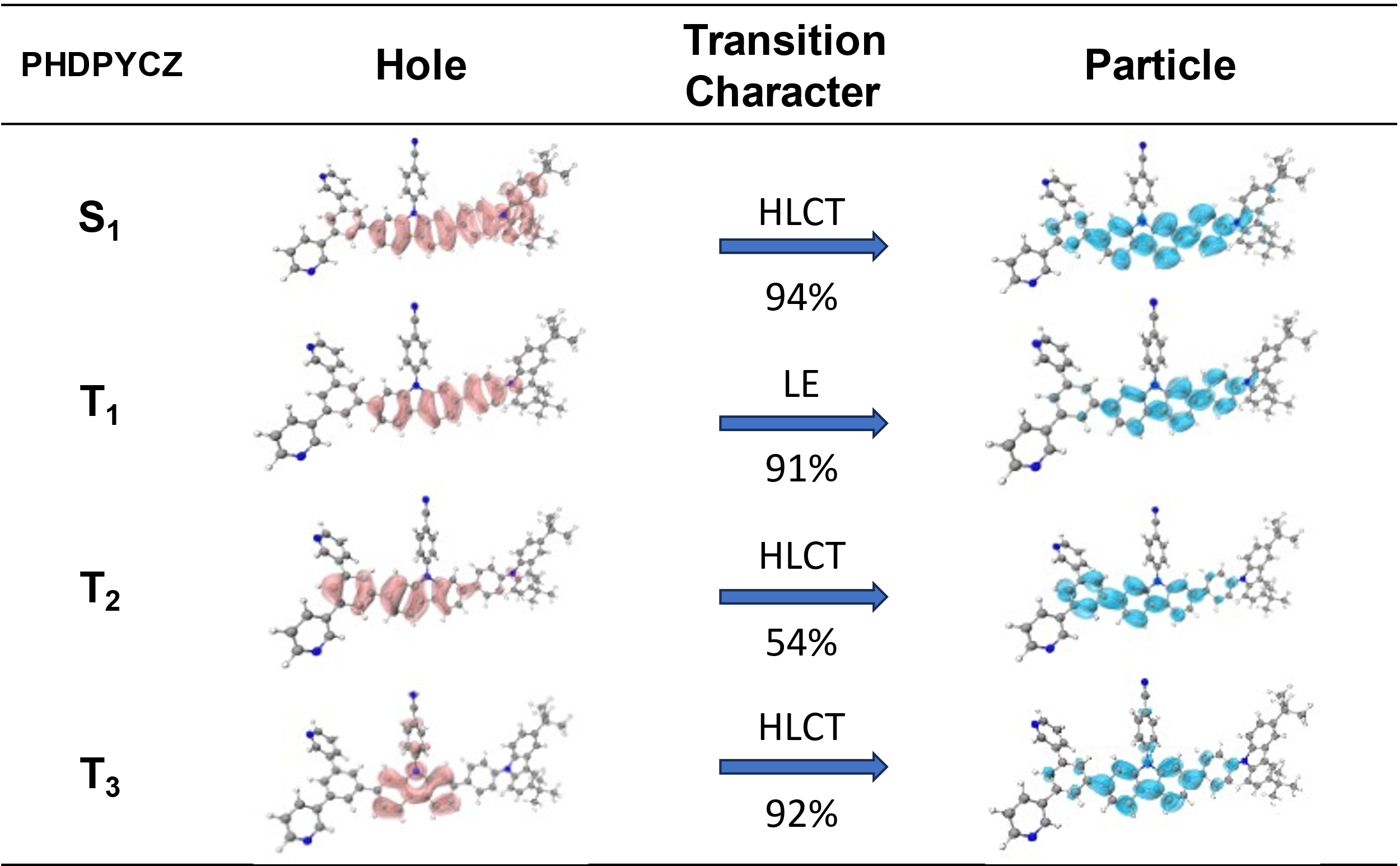


**Supplementary Figure 7** NTOs describing the transition characters of the S_1_, T_1_, T_2_ and T_3_ states in PHDPYCZ.


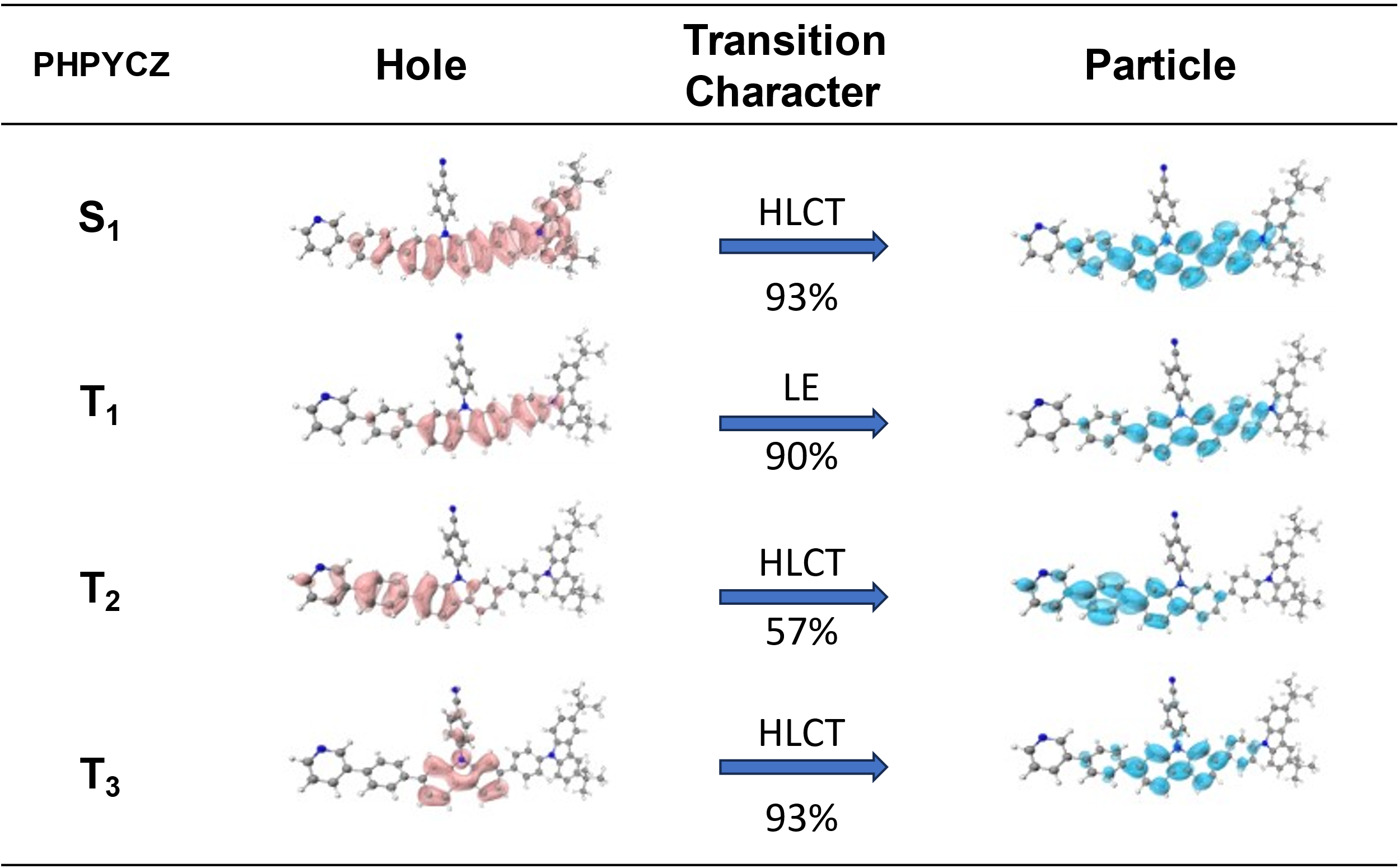


**Supplementary Figure 8** NTOs describing the transition characters of the S_1_, T_1_, T_2_ and T_3_ states in PHPYCZ.

1. **Additional Spectra and Data
   The Lippert-Mataga model**

The solvent effects on Stokes shifts are modelled by the Lippert–Mataga equation:

$hc\left( v_{a}- v_{f} \right)=hc\left( \nu_{a}^{0}-\nu_{f}^{0} \right)+ \frac{{2\left( \mu_{e}-\mu_{g} \right)}^{2}}{a_{0}^{3}}f(\varepsilon,n)$ S1

or

$\mu_{e}= \mu_{g}+{\{\frac{{hca}_{0}^{3}}{2}\cdot\left[ \frac{d\left( v_{a}- v_{f} \right)}{df\left( \varepsilon,n \right)} \right]\}}^{1/2}$ S2

where *μ*_e_ is the dipole moment of excited state, *μ*_g_ is the dipole moment of ground state, h is the Plank constant, c is the light speed in vacuum, a_0_ is the solvent Onsager cavity radius, $v_{a}- v_{f}$ is the Stokes shift, $f(\varepsilon,n)$ is the orientational polarizability of solvents and $f\left( \varepsilon,n \right)=[\frac{\varepsilon-1}{2\varepsilon+1}-\frac{n^{2}-1}{2n^{2}+1}$].$\varepsilon$ is the solvent dielectric constant and n is the solvent refractive index. *μ*_g_ was estimated by DFT. The differential $\frac{d\left( v_{a}- v_{f} \right)}{df\left( \varepsilon,n \right)}$ can be estimated based on the solvatochromic experiment data.


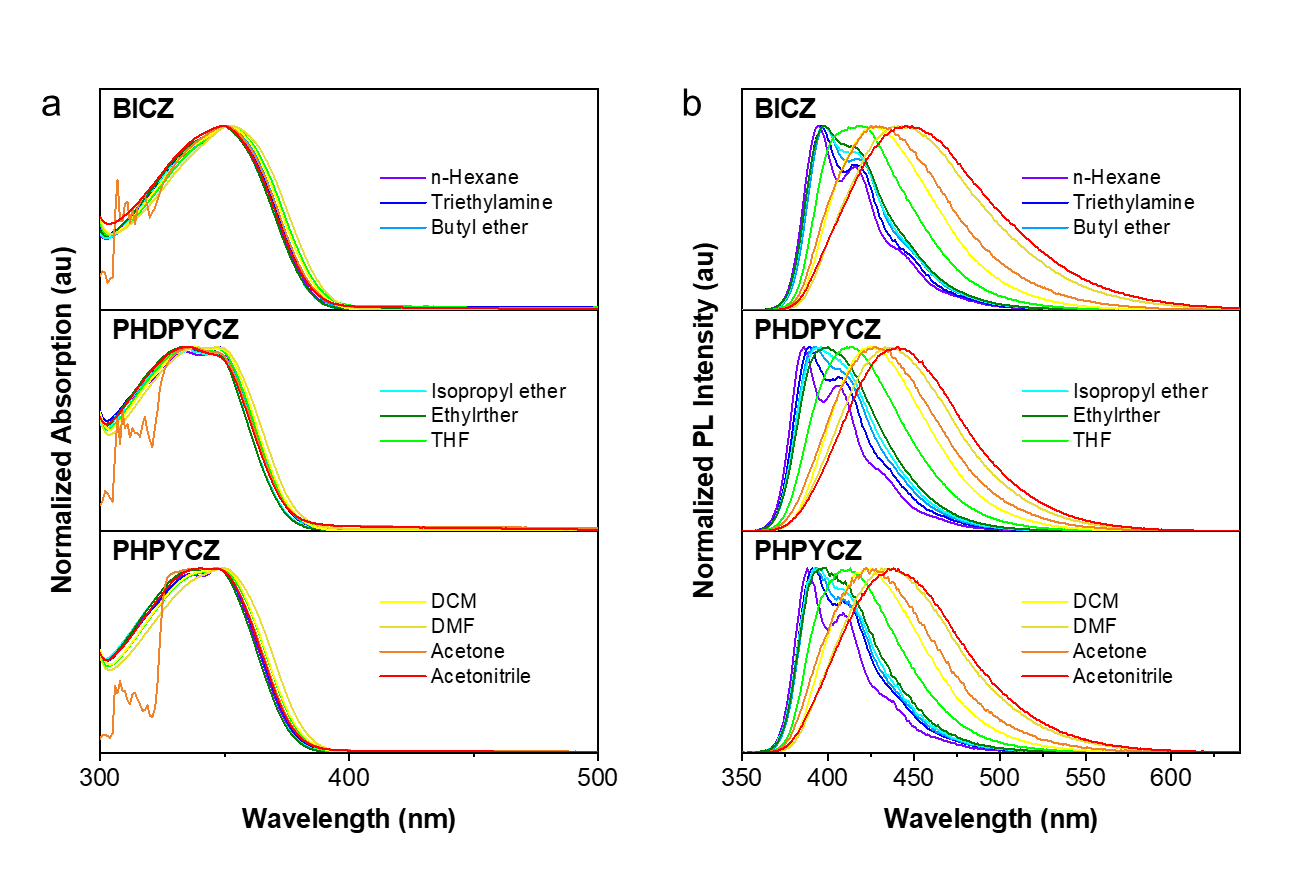


**Supplementary Figure 9** a) The solvatochromic absorption spectra and b) The solvatochromic PL spectra of BICZ, PHDPYCZ and PHPYCZ.

**Supplementary Figure 10** Fluorescence spectra in 10^−5^ toluene at RT and phosphorescence spectra in toluene at 77 K of BICZ, PHDPYCZ and PHPYCZ.

**
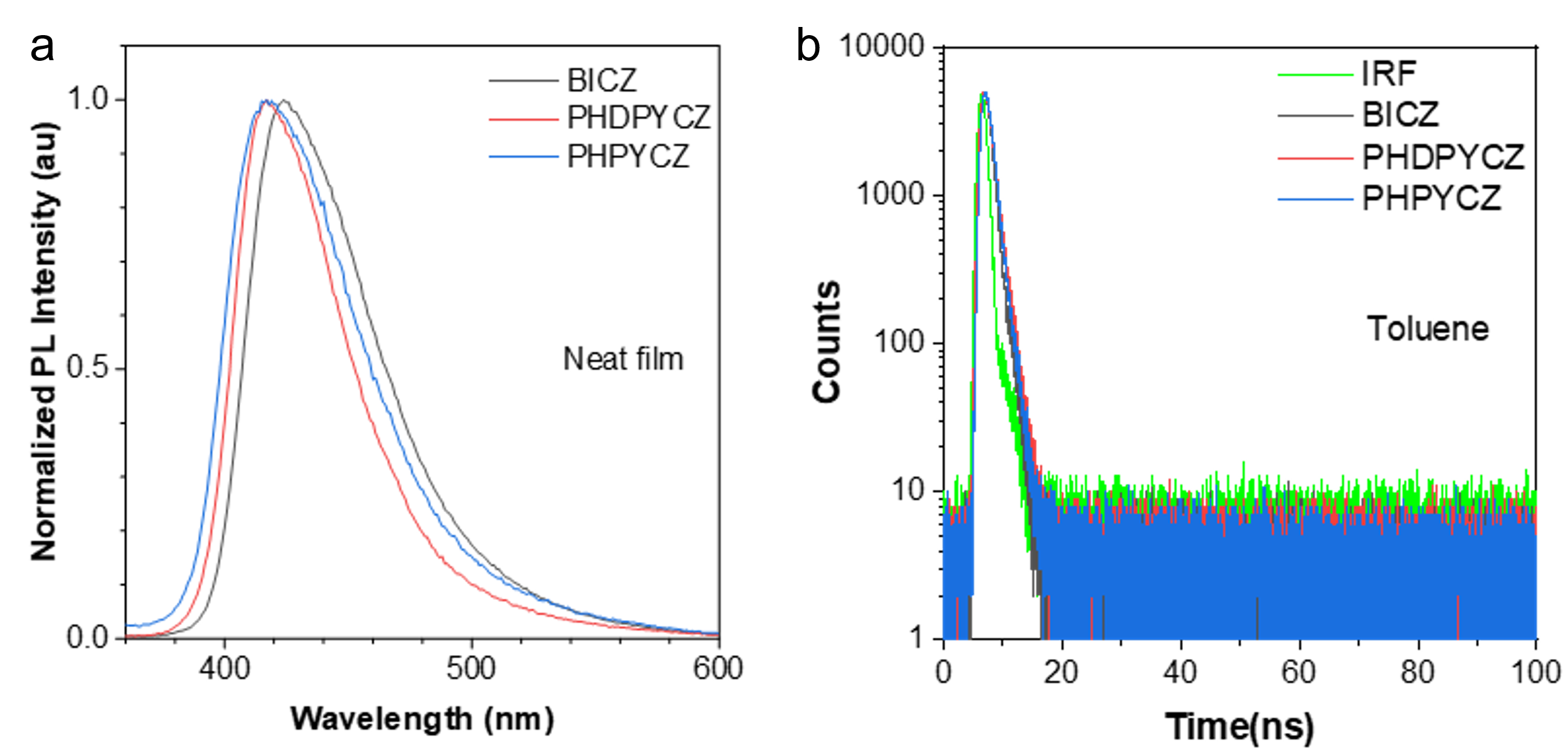
**

**Supplementary Figure 11** a) PL spectra in nest films and b) Transient PL spectra in toluene of BICZ, PHDPYCZ and PHPYCZ.

1. **Device fabrication and measurement**

The electroluminescence (EL) devices were fabricated by the vacuum-deposition method. The organic layer passes through a high-vacuum (5 × 10^−6^ Torr) onto a glass substrate pre-coated with an indium tin oxide (ITO) layer with a sheet resistance of 25 Ω square^−1^. The ITO substrates need to be soaked in ultrasonic bath of acetone, isopropanol, detergent, and deionized water respectively for 10 minutes and dried before it can be transferred to the evaporation chamber for making further efforts. The vacuum-deposited OLEDs were fabricated in the Fangsheng OMV-FS380 vacuum deposition system under a pressure of < 5 × 10^−4^ Pa. The organic films, LiF and aluminum were deposited according to the OLED configurations at deposition rates of 1~2 Å s^−1^, 0.1 Å s^−1^ and 3~5 Å s^−1^, respectively. The active area of each device was 3 mm × 3 mm.

The luminance–voltage–current density and external quantum efficiency were characterized with a dual-channel Keithley 2614B source meter and a PhotoResearch PR670 spectroradiometer. The EL spectra were obtained *via* an a Keithley 2400 Source Meter and a PhotoResearch PR670 spectroradiometer. The external quantum efficiencies were estimated utilizing the normalized EL spectra and the current efficiencies of the devices.

**
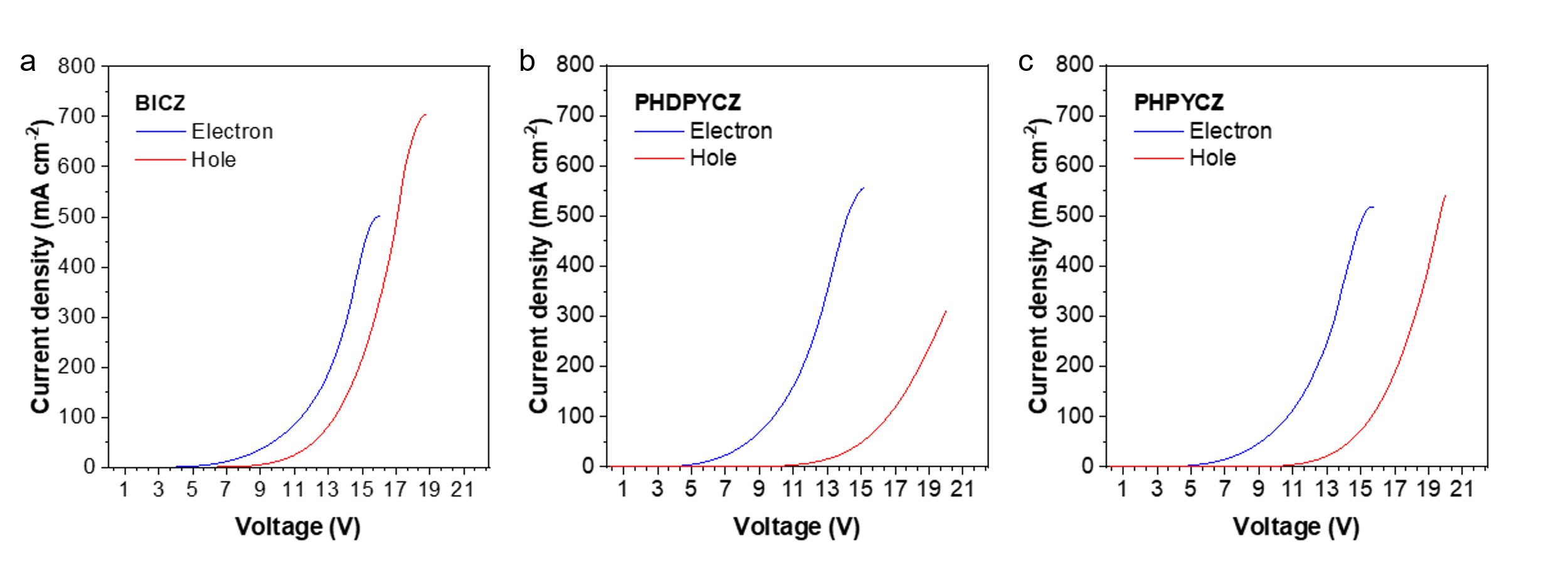
**

**Supplementary Figure 12** Plots of current density versus applied voltage of the devices.


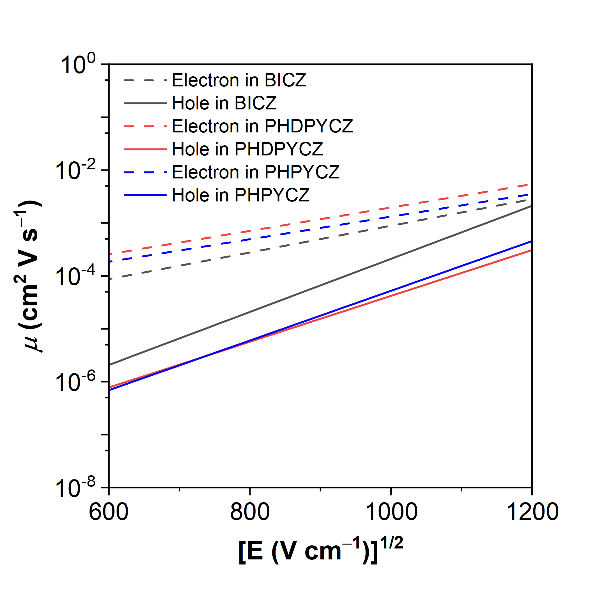


**Supplementary Figure 13** Carrier mobilities of BICZ, PHDPYCZ and PHPYCZ. At an electric field strength of 5.0 × 10^5^ V cm^-1^, the electron mobilities of BICZ, PHDPYCZ and PHPYCZ are 1.62 × 10^-4^, 4.43 × 10^-4^ and 3.13 × 10^-4^ cm^2^V^−1^s^−1^, and their hole mobilities are 7.14 × 10^-6^, 2.26 × 10^-6^ and 2.20 × 10^-6^ cm^2^V^−1^s^−1^, respectively.


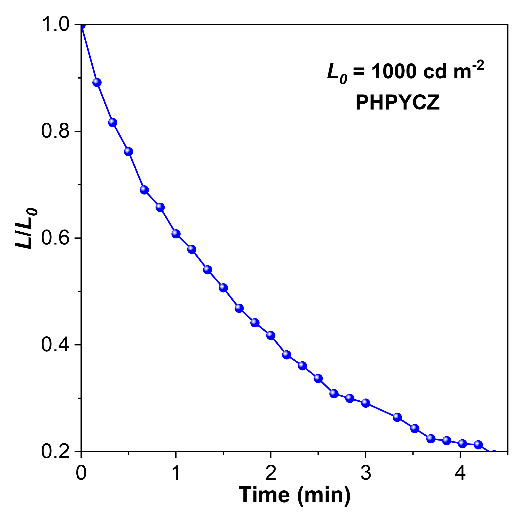


**Supplementary Figure 14** The operational lifetime of non-doped devices beaded on PHPYCZ with a configuration of ITO/HATCN (5 nm)/TAPC (25 nm)/TcTa (15 nm)/PHPYCZ (20 nm)/TmPyPB (40 nm)/LiF (1 nm)/Al. The lifetime of decay to 50% of the initial brightness at 1000 brightness is only 1.5 minutes.


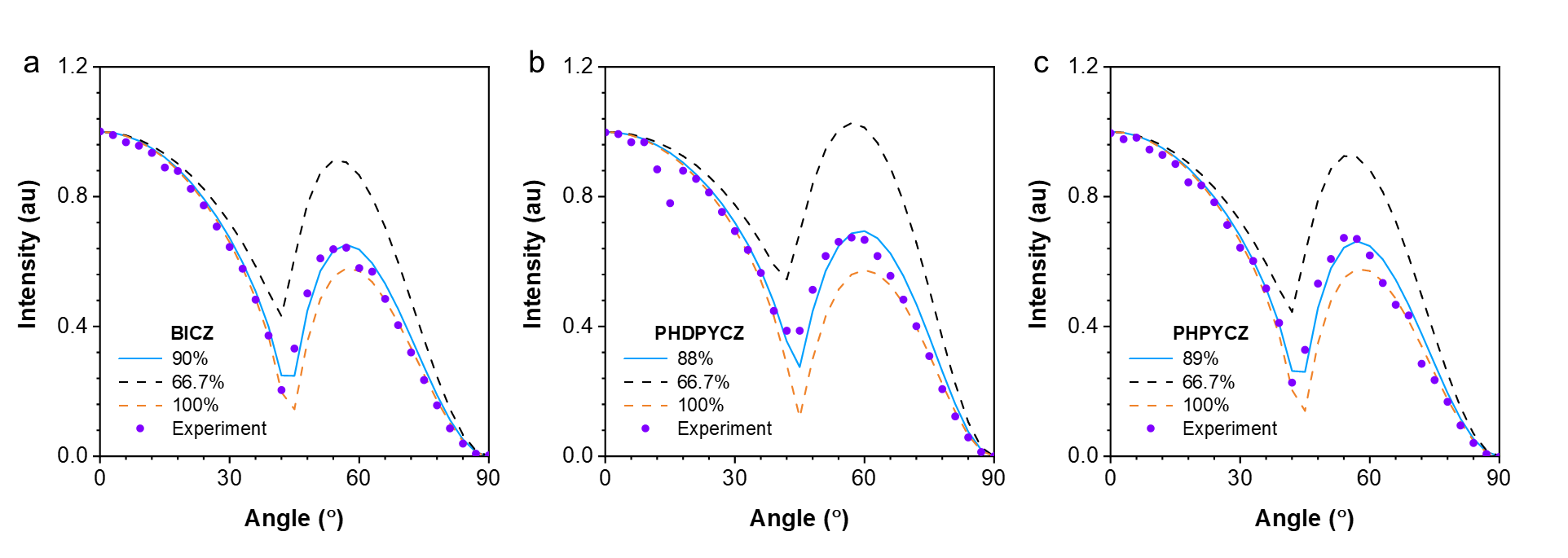


**Supplementary Figure 15** Measured the angle-dependent p-polarized PL spectra of (a) BICZ, (b) PHDPYCZ and (c) PHPYCZ in non-doped film.

**
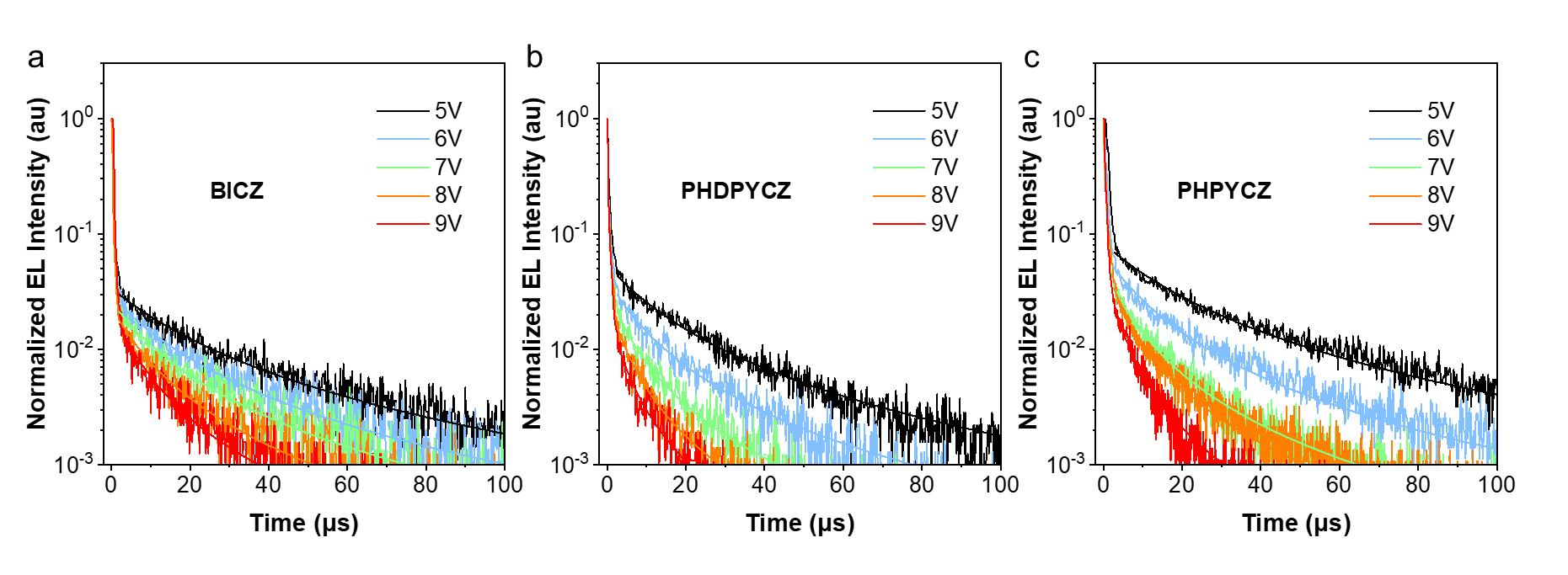
**

**Supplementary Figure 16** Transient EL data fitted with the TTA model.

^
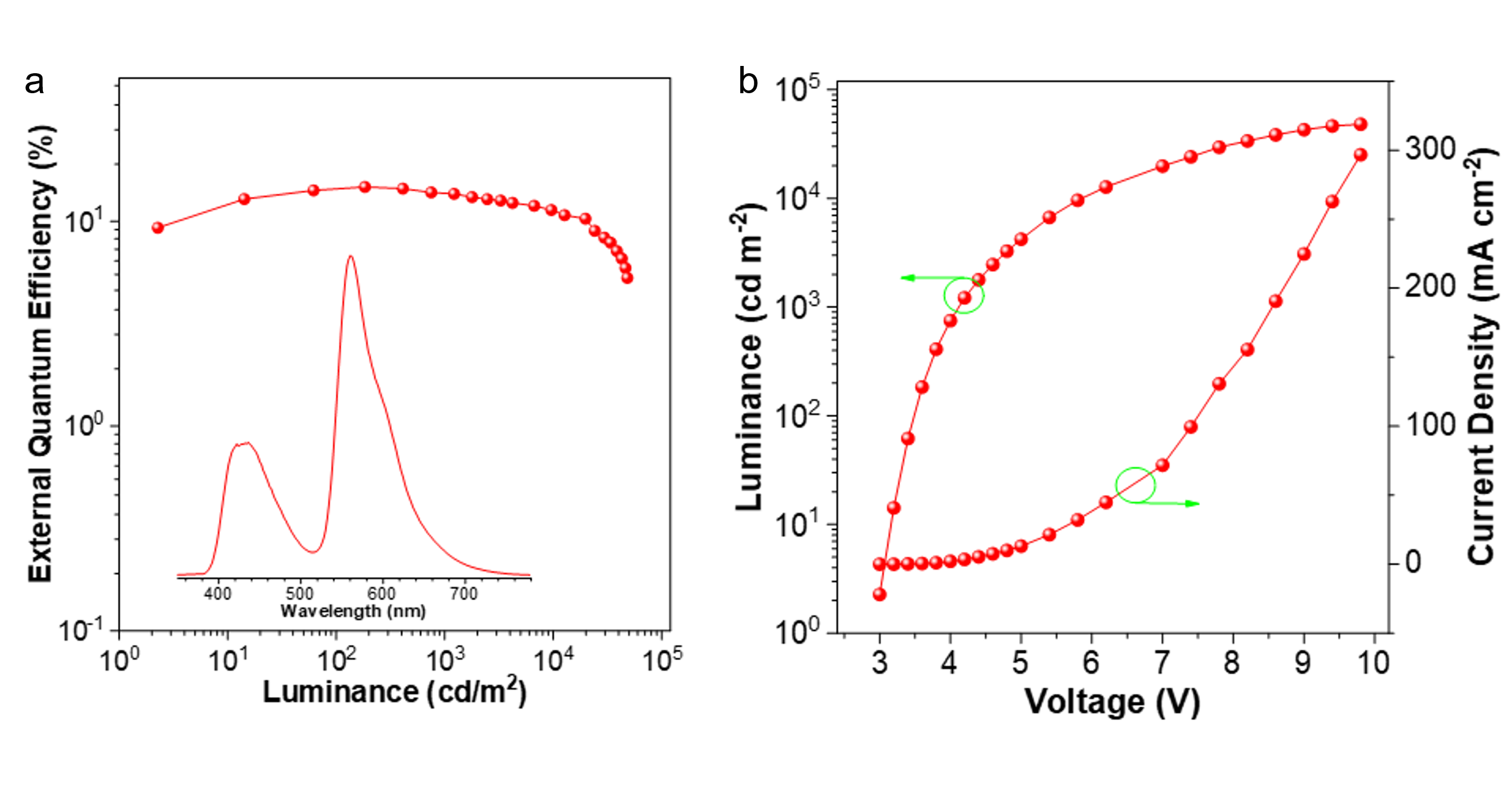
^

**Supplementary Figure 17** The two-color hybrid white OLED with a configuration of ITO/HATCN (5 nm)/TAPC (25 nm)/TcTa (15 nm)/PHPYCZ (10 nm)/PHPYCZ: 3 wt% PO-01 (8 nm)/TmPyPB (40 nm)/LiF (1 nm)/Al. We used PHPYCZ to fabricate two-color hybrid WOLED, where the neat film of PHPYCZ was used as a deep-blue EML and the layer of PHPYCZ: 3 wt% PO-01-TB is used as an orange EML. This device shows excellent white light emission with the CIE coordinate of (0.380,0.381), and the values of η_c,max_, η_p,max_, and η_ext,max_ are 38.66 cd A^−1^, 34.37 lm W^−1^, and 14.71 %, respectively. Moreover, this device can maintain η_ext_ of 13.82% at 1000 cd m^−2^, respectively, exhibiting a small efficiency roll-off under practical lighting luminance.

**
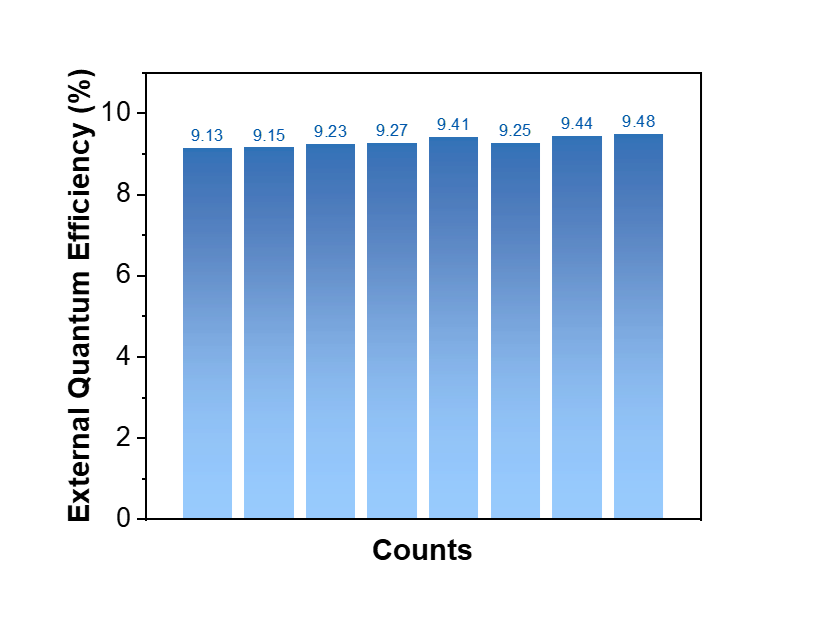
**

**Supplementary Figure 18** Repetition of external quantum efficiency. 8 non-doped devices of PHPYCZ were measured.

1. **Configuration optimization of non-doped device based on PHPYCZ**

**Device Configuration:**

1) ITO/HATCN (5 nm)/TAPC (25 nm)/TcTa (15 nm)/ PHPYCZ (20 nm)/TmPyPB (40 nm)/ LiF (1 nm)/Al.

2) ITO/HATCN (5 nm)/TAPC (25 nm)/TcTa (15 nm)/ PHPYCZ (20 nm)/TmPyPB (30 nm)/ LiF (1 nm)/Al.

3) ITO/HATCN (5 nm)/TAPC (25 nm)/TcTa (15 nm)/ PHPYCZ (20 nm)/TmPyPB (25 nm)/ LiF (1 nm)/Al.

| Device | λ_EL_  [nm] | FWHM  [nm] | *V*_on_  [V] | *L*_max_  [cd/m^2^] | *η*_c_  [cd/A] | *η*_p_  [lm/W] | EQEmax/  EQE@1000 cd m^-2^ | CIE (x, y) |
| --- | --- | --- | --- | --- | --- | --- | --- | --- |
| 1# | 418 | 58 | 3.4 | 7917 | 3.49 | 2.86 | 9.48/8.05 | (0.154,0.049) |
| 2# | 418 | 58 | 3.2 | 7969 | 3.10 | 2.58 | 9.44/7.92 | (0.155,0.044) |
| 3# | 418 | 58 | 3.2 | 7209 | 2.83 | 2.36 | 9.28/7.95 | (0.155,0.041) |

**Device Configuration:**

1) ITO/HATCN (5 nm)/TAPC (25 nm)/TcTa (15 nm)/ PHPYCZ (20 nm)/TPBi (40 nm)/ LiF (1 nm)/Al.

2) ITO/HATCN (5 nm)/TAPC (50 nm)/TcTa (5 nm)/ PHPYCZ (20 nm)/TPBi (40 nm)/ LiF (1 nm)/Al.

| Device | λ_EL_  [nm] | FWHM  [nm] | *V*_on_  [V] | *L*_max_  [cd/m^2^] | *η*_c_  [cd/A] | *η*_p_  [lm/W] | EQEmax/  EQE@1000 cd m^-2^ | CIE (x, y) |  |
| --- | --- | --- | --- | --- | --- | --- | --- | --- | --- |
| 1# | 418 | 56 | 3.4 | 7978 | 2.20 | 1.87 | 6.29/6.06 | (0.155,0.048) |  |
| 2# | 418 | 54 | 3.6 | 6997 | 2.34 | 1.84 | 6.92/6.44 | (0.156,0.048) |  |

**Device Configuration:**

1): ITO/HATCN (5 nm)/TAPC (50 nm)/TcTa (5 nm)/ mcp (5 nm)/ PHPYCZ (20 nm)/ PPF (5 nm)/ TmPyPB (40 nm)/ LiF (1 nm)/Al.

2): ITO/HATCN (5 nm)/TAPC (50 nm)/TcTa (5 nm)/ mcp (5 nm)/ PHPYCZ (20 nm)/ PPF (5 nm)/ TPBi (40 nm)/ LiF (1 nm)/Al.

| Device | λ_EL_  [nm] | FWHM  [nm] | *V*_on_  [V] | *L*_max_  [cd/m^2^] | *η*_c_  [cd/A] | *η*_p_  [lm/W] | EQEmax/  EQE@1000 cd m^-2^ | CIE (x, y) |
| --- | --- | --- | --- | --- | --- | --- | --- | --- |
| 1# | 418 | 48 | 3.4 | 6499 | 2.19 | 1.66 | 6.70/6.21 | (0.159,0.047) |
| 2# | 416 | 48 | 4.0 | 5108 | 2.03 | 1.37 | 6.79/5.86 | (0.158,0.044) |

**Supplementary Table 5** Summary of EL performance of recently reported high-performance non-doped OLEDs that approaching the BT.2020 blue standard.

| Emitters | *V*_on_  [V] | *η*_c_  [cd/A] | *η*_p_  [lm/W] | *η*_ext_  [%] | *L*_max_  [cd/m^2^] | λ_EL_  [nm] | CIE (x, y) | Ref. |
| --- | --- | --- | --- | --- | --- | --- | --- | --- |
| PHPYCZ | 3.4 | 3.49 | 2.86 | 9.48 | 7917 | 418 | (0.154,0.049) | This work |
| PHDPYCZ | 3.4 | 1.95 | 1.47 | 5.17 | 4348 | 414 | (0.159,0.056) | This work |
| 3,6-mPPICNC3 | 2.9 | 1.52 | 1.35 | 7.67 | 7844 | 412 | (0.160, 0.032) | 1 |
| CZ-BO | 4.4 | 1.2 | 1 | 5.5 | − | 412 | (0.163,0.034) | 1 |
| DTPCZPHTZ | 3.2 | 1.6 | 1.7 | 5.7/5.4/- | 5866 | 427 | (0.157,0.037) | 2 |
| 2MCz-CNMCz | 3.0 | 1.76 | 1.72 | 7.76/5.61 | 4757 | 404 | (0.158, 0.039) | 3 |
| TPBCzC1 | 3.4 | 0.95/0.87 | 0.85/0.47 | 4.34/3.90 | − | 422 | (0.160, 0.035) | 4 |
| PIPDMePBO | 3.1 | 3.1 | 2.9 | 8.0/7.2/7.0 | 12212 | 427 | (0.156,0.048) | 5 |
| TPA-TAZ | − | − | − | 6.8 | − | 428 | (0.158,0.043) | 6 |
| 2FPPIDPA | 2.8 | 2.58/1.72 | 2.83/0.93 | 6.49/4.36 | 4916 | 430 | (0.156,0.046) | 7 |
| SBF-PISBF | 3.5 | 2.65 | 2.23 | 6.19/5.75/- | 3775 | 436 | (0.155,0.049) | 8 |
| 2BuCz-CNCz | − | − | − | 5.24% | − | 408 | (0.157, 0.050) | 9 |
| TTP-TPI | 3.1 | 2.10/1.47 | 1.88/0.81 | 5.02/3.98 | − | 424 | (0.16,0.05) | 10 |
| DPM | 3.1 | 1.77 | 1.39 | 4 | − | 428 | (0.157,0.053) | 11 |
| 2M-ph-pCzAnBzt | 3.4 | 5.95 | 5.43 | 10.44 | 3085 | 450 | (0.151,0.057) | 12 |
| FIP-CZ | 3.3 | 2.86 | 2 | 9.5 | 9388 | 402 | (0.170, 0.058) | 13 |
| 2PPIAn | 3 | 5.1 | 5.3 | 8.9/3.9 | − | 444 | (0.15, 0.06) | 14 |
| CSiTPI | 3.2 | 1.39 | 1.28 | 7.1/5.3 | 2445 | 404 | (0.16, 0.06) | 15 |
| CSP-OMe | 3.3 | 2.53/2.35 | 2.13/2.32 | 6.60/6.15 | 7979 | 414 | (0.157,0.058) | 16 |
| TPBPPI-PBI | 3.9 | 3.22 | 2.71 | 5.94 | − | 429 | (0.160,0.059) | 17 |
| BBPA | 3.2 | − | − | 5.28/1.35 | − | 432 | (0.15, 0.06) | 18 |

1. **NMR Spectroscopies and Mass spectrums**


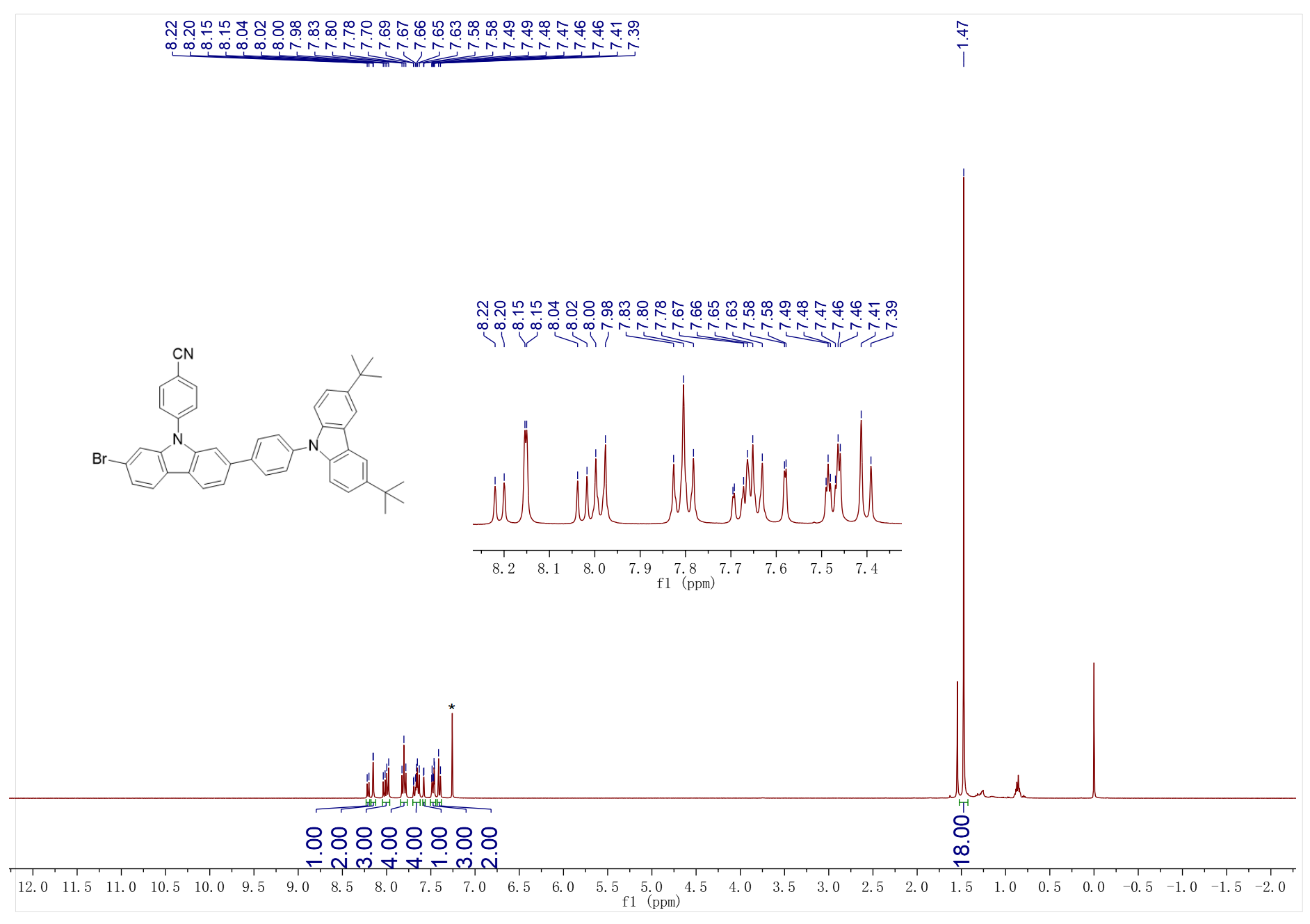


**Supplementary Figure 19** ^1^H NMR spectrum of **Br-CNCz-BuCz** in CDCl_3_.


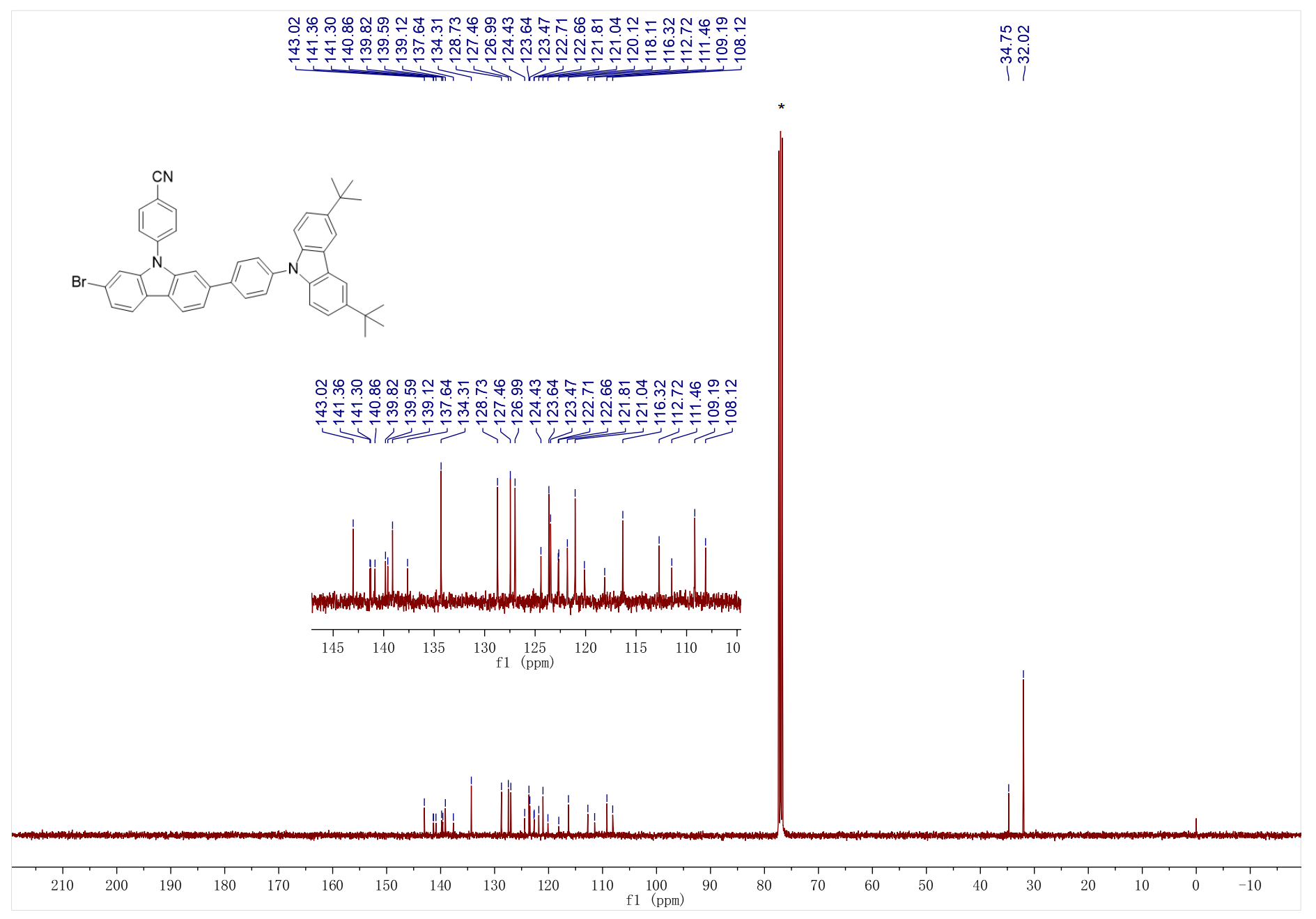


**Supplementary Figure 20** ^13^C NMR spectrum of **Br-CNCz-BuCz** in CDCl_3_.


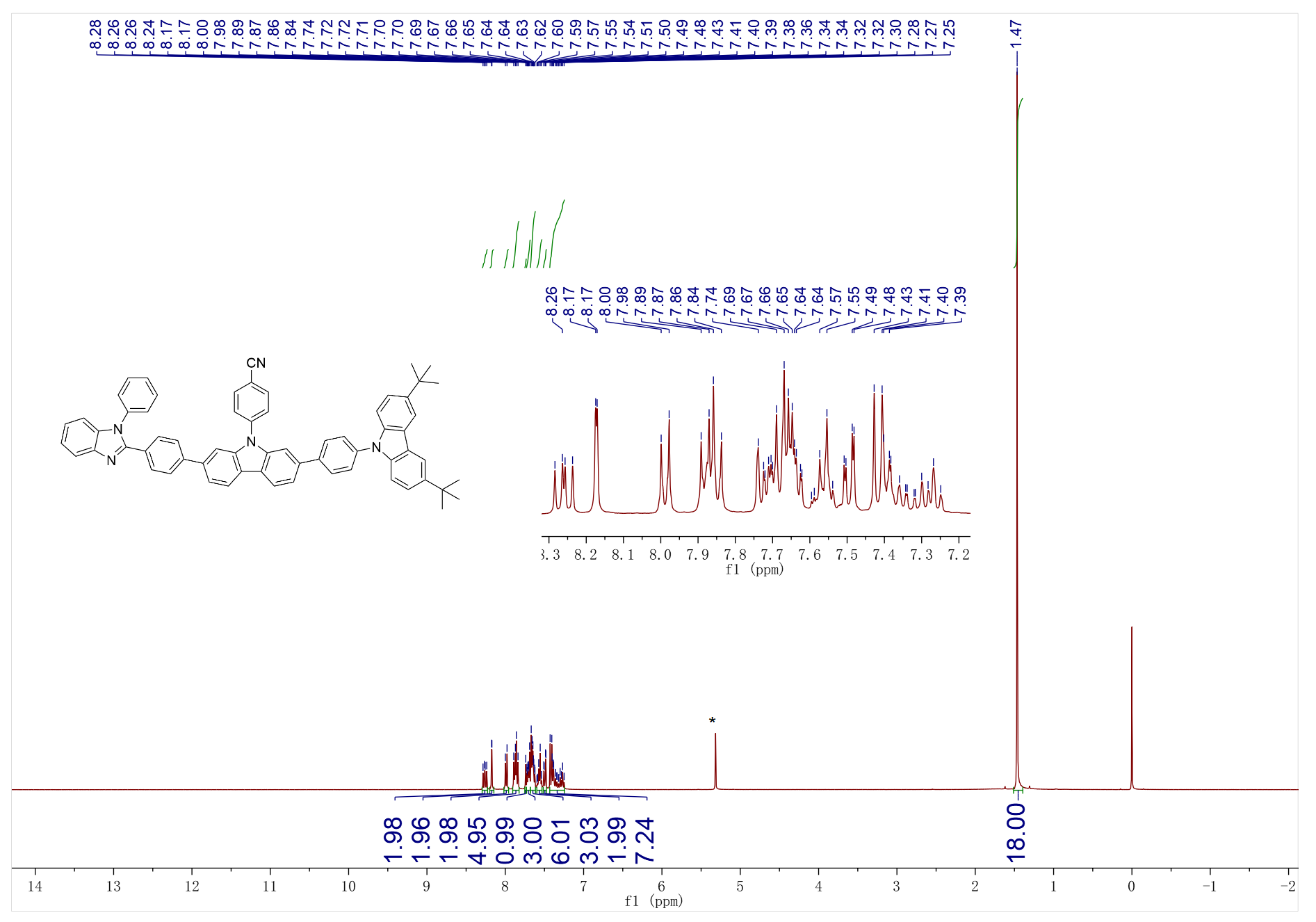


**Supplementary Figure 21** ^1^H NMR spectrum of **BICZ** in CD_2_Cl_2_.


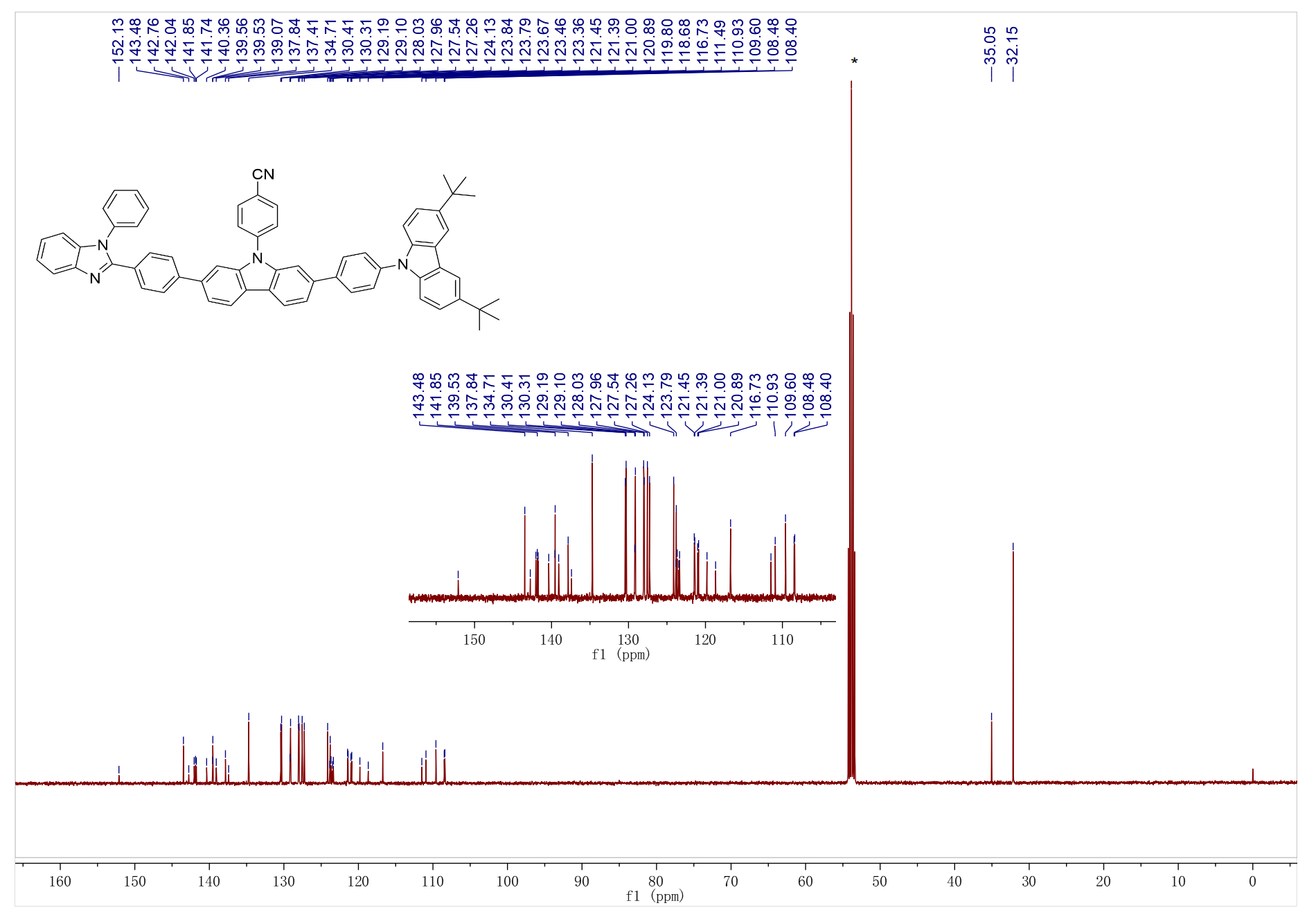


**Supplementary Figure 22** ^13^C NMR spectrum of **BICZ** in CD_2_Cl_2_.


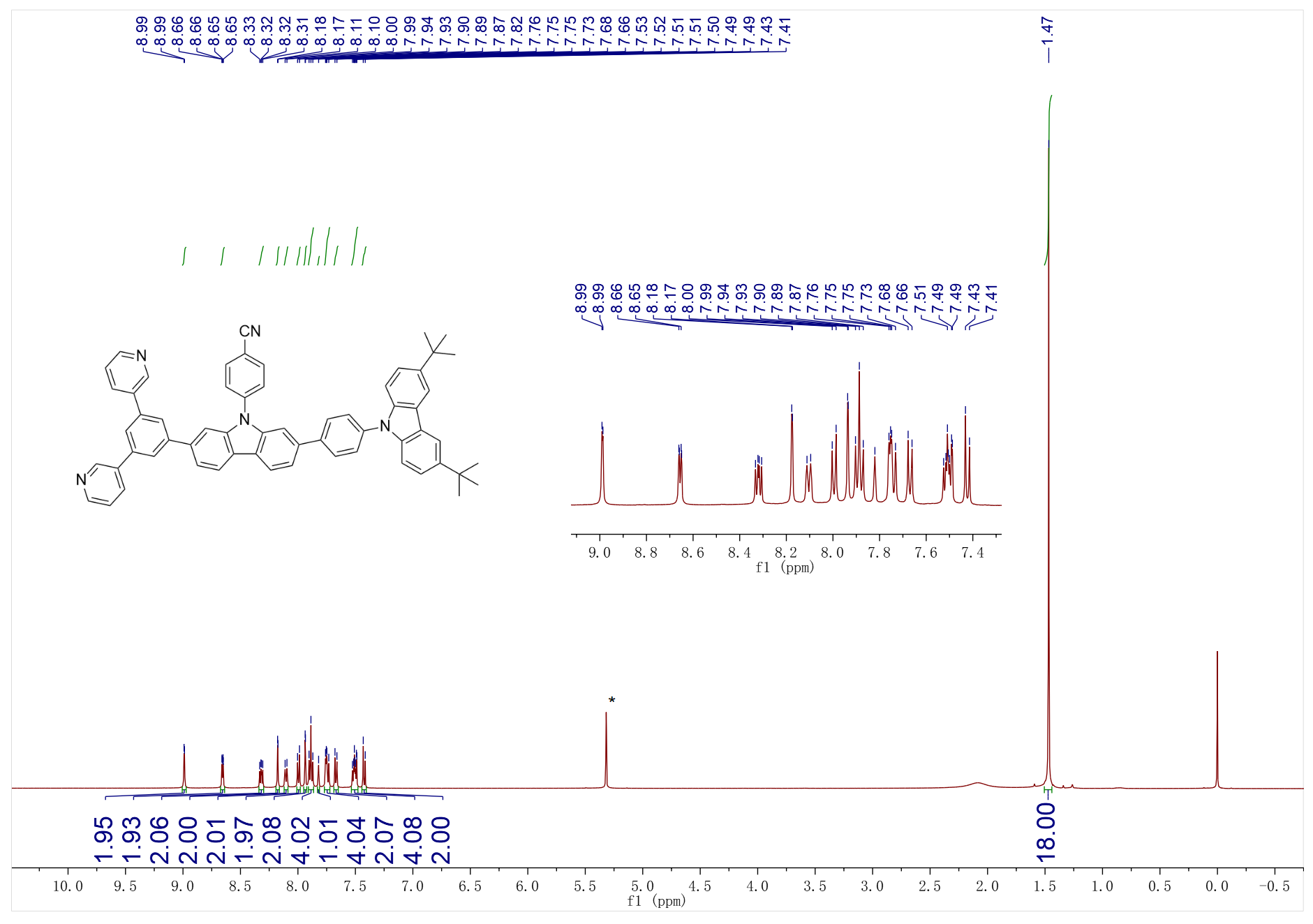


**Supplementary Figure 23**  ^1^H NMR spectrum of **PHDPYCZ** in CD_2_Cl_2_.


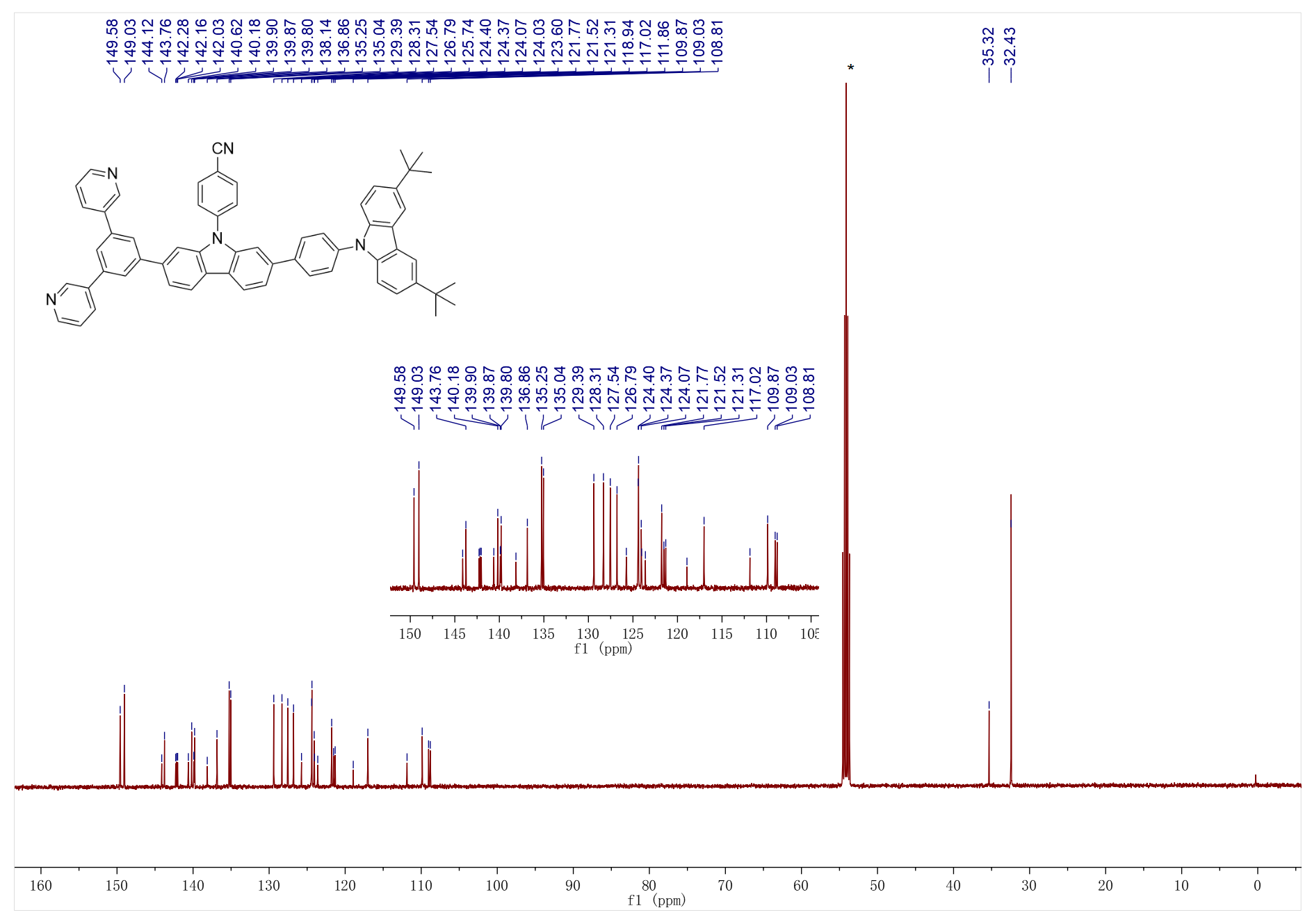


**Supplementary Figure 24** ^13^C NMR spectrum of **PHDPYCZ** in CD_2_Cl_2_.


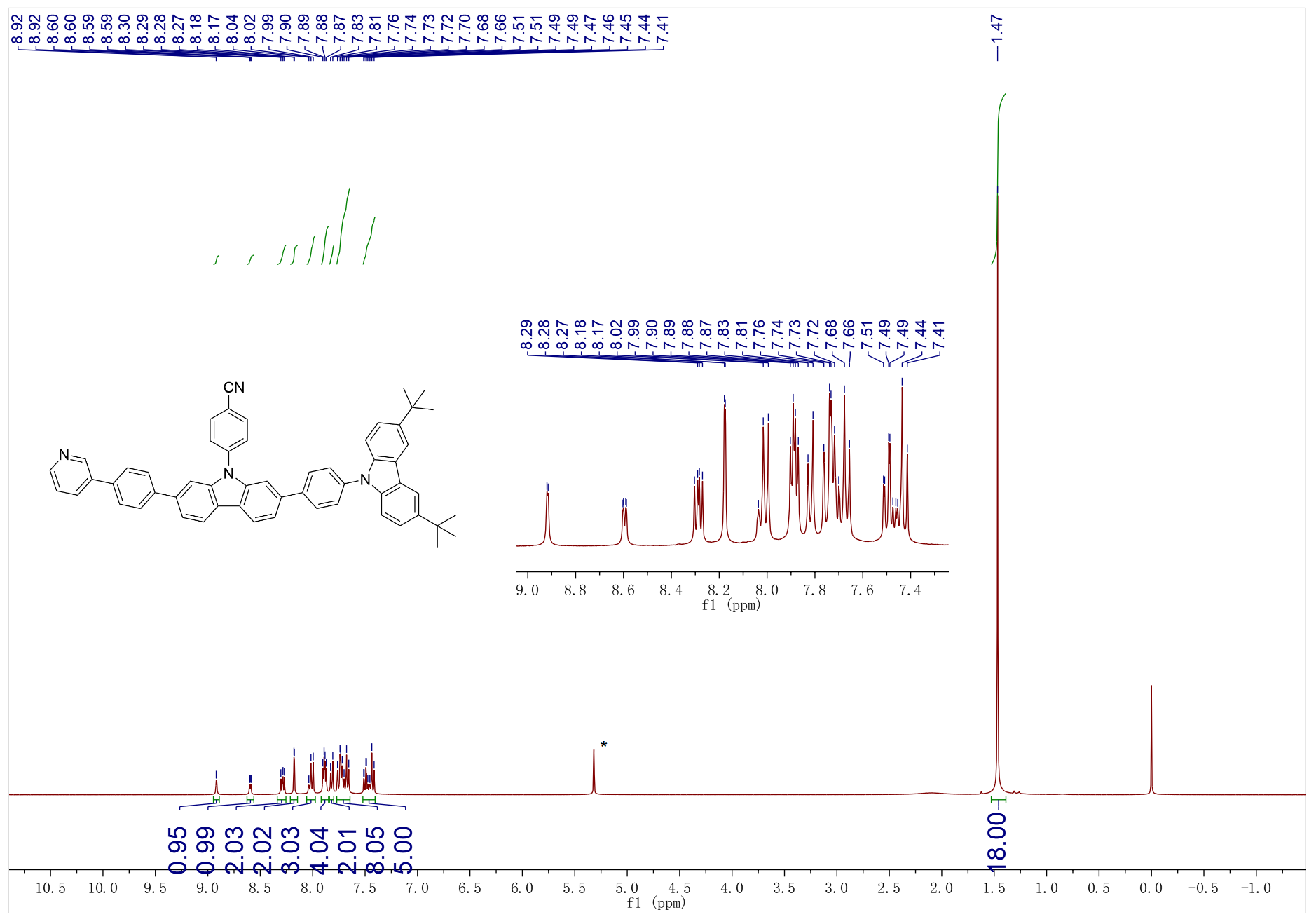


**Supplementary Figure 25** ^1^H NMR spectrum of **PHPYCZ** in CD_2_Cl_2_.


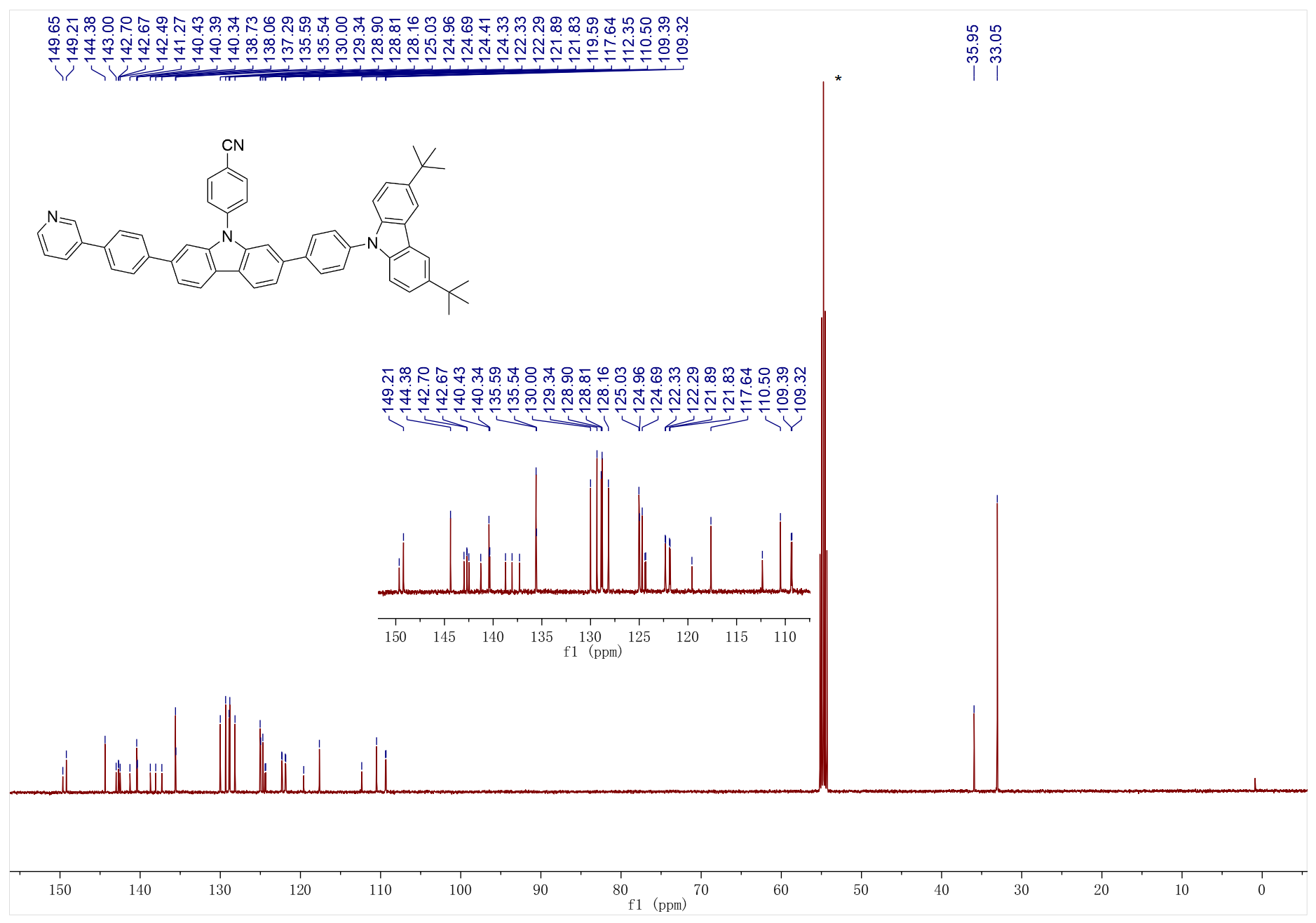


**Supplementary Figure 26** ^13^C NMR spectrum of **PHPYCZ** in CD_2_Cl_2_.


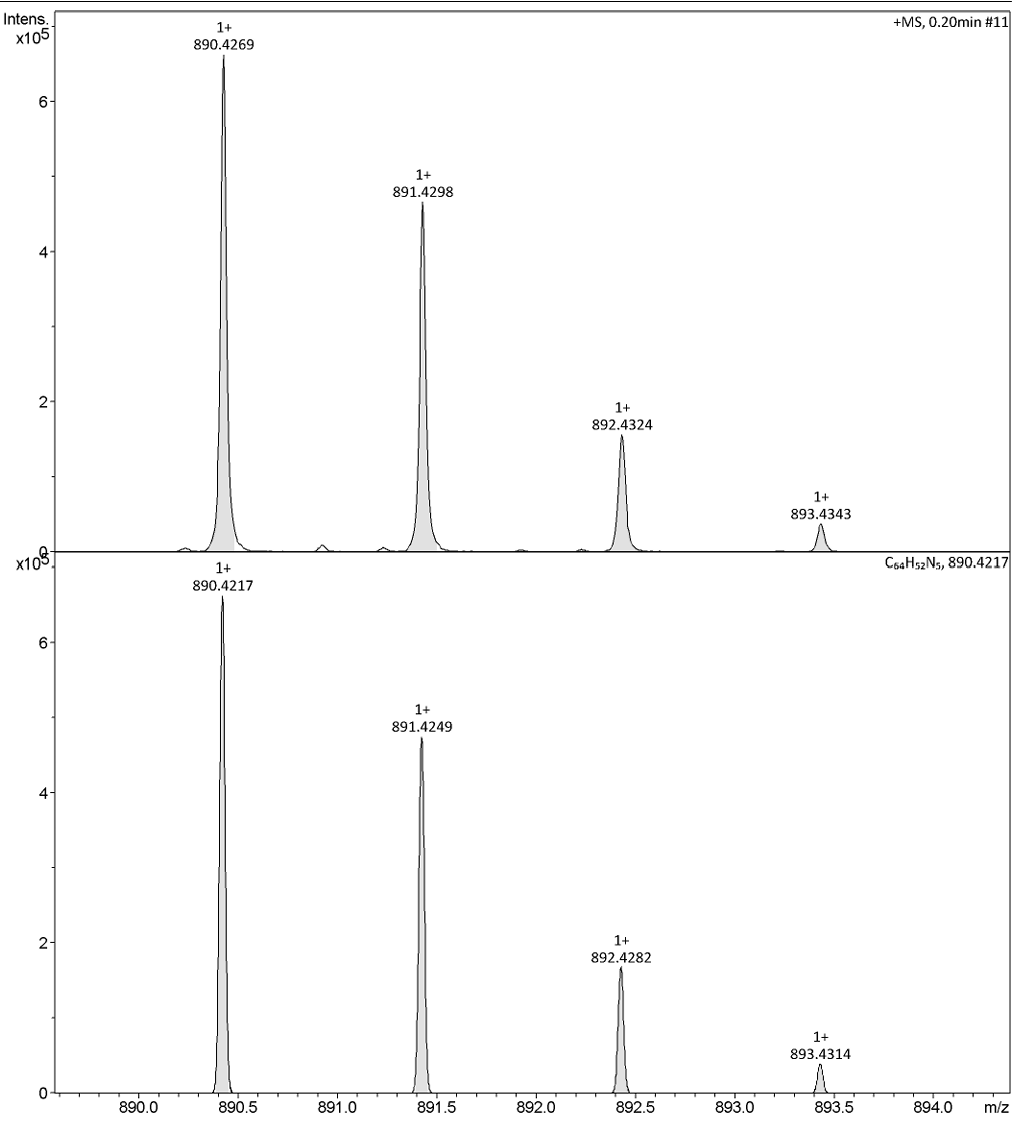


**Supplementary Figure 27** High resolution mass spectra (HRMS) of **BICZ.**


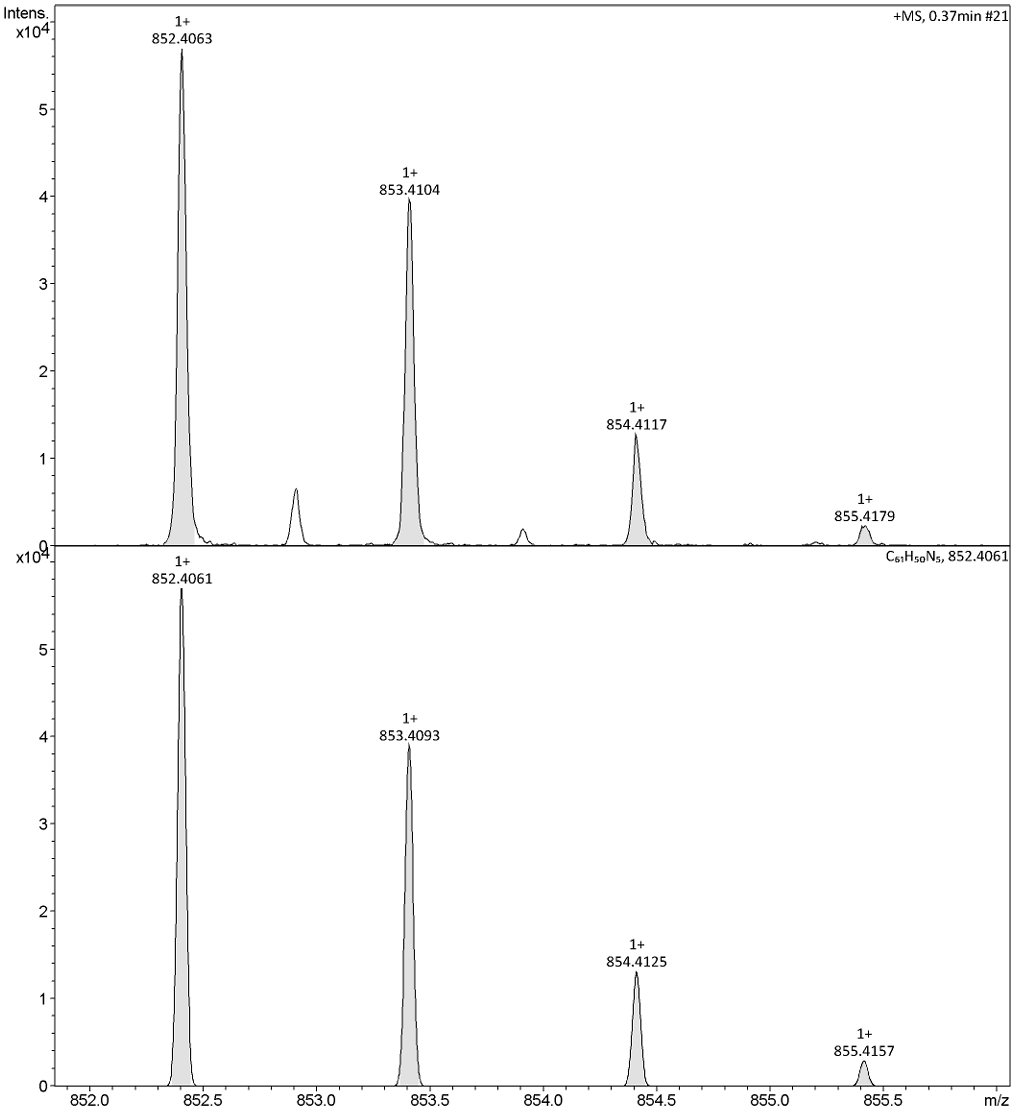


**Supplementary Figure 28** High resolution mass spectra (HRMS) of **PHDPYCZ.**

**
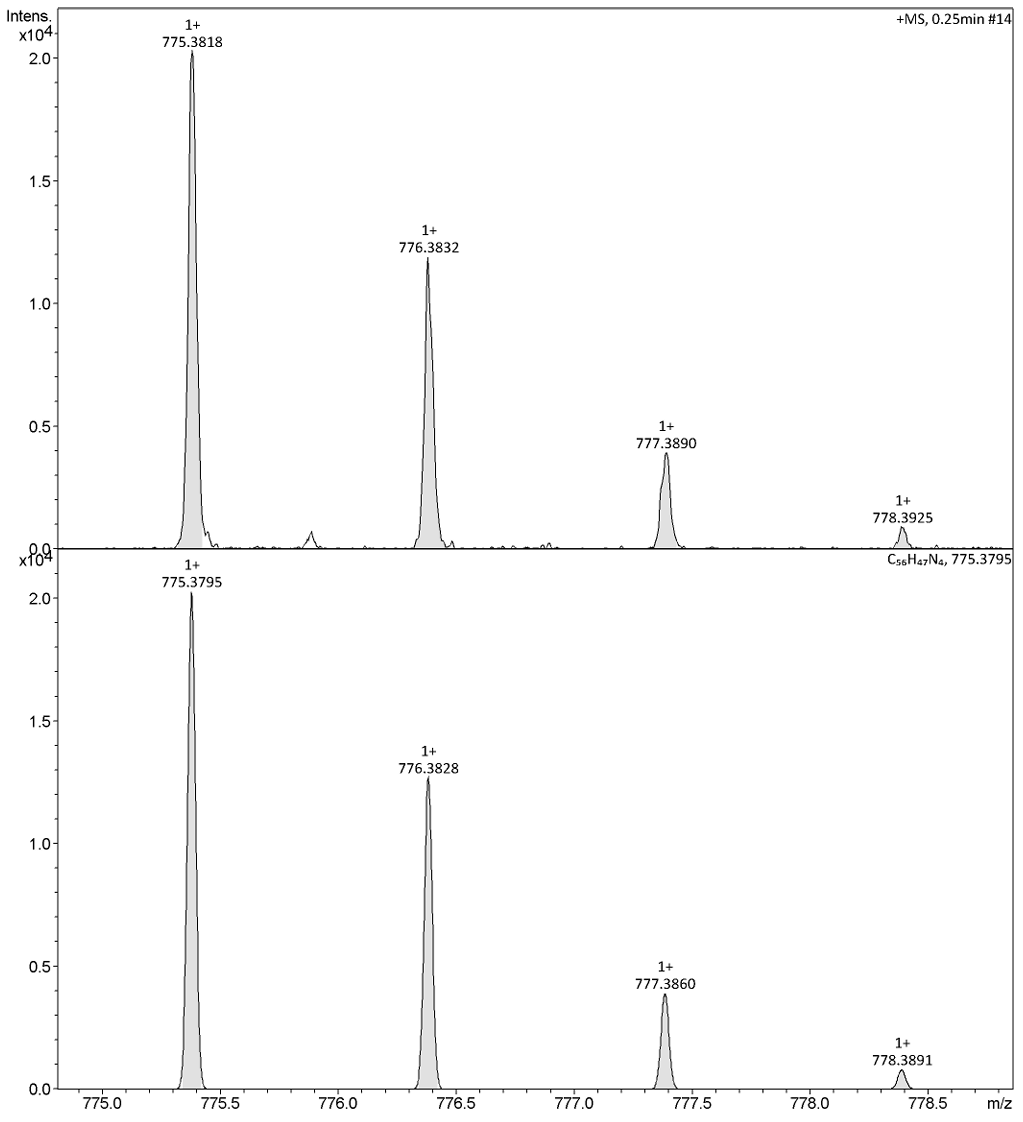
**

**Supplementary Figure 29** High resolution mass spectra (HRMS) of **PHPYCZ.**

**References:**

[1] H. Qi, D. Xie, Z. Gao, S. Wang, L. Peng, Y. Liu, S. Ying, D. Ma, S. Yan, *Chem. Sci.* **2024**, Accepted Manuscript.

[2] Z. Zhao, C. Zeng, X. Peng, Y. Liu, H. Zhao, L. Hua, S. J. Su, S. Yan, Z. Ren, *Angew. Chem. Int. Ed.* **2022**, 61, e202210864.

[3] M. Sun, T. Li, M. Xie, H. Zhou, Q. Sun, D. Liu, Y. Pan, S. Zhang, W. Yang, S. Xue, *Dyes and pigments* **2023**, *210*, 111002.

[4] X. Guo, G. Li, J. Lou, K. Chen, R. Huang, D. Yang, H. Zhang, Z. Wang, B. Z. Tang, *Small* **2022**, *18*, 2204029.

[5] P. Han, C. Lin, D. Ma, A. Qin, B. Z. Tang, *ACS Appl. Mater. Interfaces* **2020**, 12, 46366.

[6] C. Liu, Y. Zhou, T. Li, H. Zhou, M. Xie, L. Chu, Q. Sun, S. Zhang, W. Yang, S. Xue, *Chemical Engineering Journal* **2023**, *471*, 144505.

[7] A. Obolda, Q. Peng, C. He, T. Zhang, J. Ren, H. Ma, Z. Shuai, F. Li, *Adv Mater* **2016**, *28*, 4740.

[8] Z. Li, N. Xie, Y. Xu, C. Li, X. Mu, Y. Wang, *Organic Materials* **2020**; 02, 011.

[9] S. S. Tang, G. X. Yang, J. J. Zhu, X. He, J. X. Jian, F. Lu, Q. X. Tong, *Chemistry* **2021**, *27*, 9102

[10] H. Zhang, G. Li, X. Guo, K. Zhang, B. Zhang, X. Guo, Y. Li, J. Fan, Z. Wang, D. Ma, B. Z. Tang, *Angew. Chem. Int. Ed.* **2021**, *60*, 22241.

[11] Y. Yuan, J.-. Chen, F. Lu, Q.-X. Tong, Q.-D. Yang, H.-W. Mo, T.-W. Ng, F.-L. Wong, Z.-Q. Guo, J. Ye, Z. Chen, X.-H. Zhang, C.-S. Lee, *Chem. Mater.* **2013**, 25, 4957.

[12] S. Xiao, S. Zhang, Y. Gao, X. Yang, H. Liu, W. Li, B. Yang, *Dyes and pigments* **2021**, *193*, 109482.

[13] R. Guo, W. Liu, S. Ying, Y. Xu, Y. Wen, Y. Wang, D. Hu, X. Qiao, B. Yang, D. Ma, L. Wang, *Science bulletin* **2021**, *66*, 2090.

[14] C. Liao, B. Chen, Q. Xie, X. Li, H. Liu, S. Wang, *Advanced Materials* **2023**, *35*.

[15] J. S. Huh, Y. H. Ha, S. K. Kwon, Y. H. Kim, J. J. Kim, *ACS Appl Mater Interfaces* **2020**, *12*, 15422.

[16] Y. Zheng, X. Zhu, Z. Ni, X. Wang, Z. Zhong, X. J. Feng, Z. Zhao, H. Lu, *Advanced Optical Materials* **2021**, 9, 2100965.

[17] S. Geng, Z. Liu, H. Li, Z. Zhong, X. J. Feng, Z. Zhao, H. Lu, *Advanced optical materials* **2024**, *12*.

[18] J.-J. Zhu, W.-C. Chen, Y. Yuan, D. Luo, Z.-L. Zhu, X. Chen, J.-X. Chen, C.-S. Lee, Q.-X. Tong, *Dyes and pigments* **2020**, 173, 107982.

[19] S. Wang, M. Qiao, Z. Ye, D. Dou, M. Chen, Y. Peng, Y. Shi, X. Yang, L. Cui, J. Li, C. Li, B. Wei, W. Y. Wong, *iScience* **2018**, *9*, 532.
